# Sustainable Hydrogel‐Forming Microneedle‐Reservoir Patches for Transdermal Delivery of Hydrophilic Macromolecules: Biopolymers, Green Manufacturing and Translation

**DOI:** 10.1002/advs.78195

**Published:** 2026-10-11

**Authors:** Siddharth Mohanty, Mehmet Onur Aydogdu, Sijia Kang, Xinbo Li, Mohan Edirisinghe

**Affiliations:** ^1^ Department of Mechanical Engineering University College London (UCL) London UK

**Keywords:** biopolymers, fluorescein isothiocyanate‐dextran, hydrogel‐forming microneedles, hydrophilic macromolecules, microneedle‐reservoir patches, pressurized gyration, sustainability, transdermal drug delivery

## Abstract

Hydrophilic biomacromolecules, including insulin, peptides, proteins and vaccine antigens, have transformed modern therapeutics, yet their delivery remains dominated by injections because the stratum corneum (SC) severely restricts passive transport of large and polar molecules. Microneedles have created a route through this barrier, but many existing systems remain limited by low loading capacity, burst release, weak dose control, microbiological quality requirements, short‐term stability, and poor manufacturing scalability. This review critically examines hydrogel‐forming microneedle (HFMN)‐reservoir patches as an emerging architecture for controlled transdermal biomacromolecule delivery. Rather than treating microneedles as isolated projections, it positions the patch as an integrated multilayer device comprising a skin‐interfacing hydrogel conduit, a drug reservoir, a diffusion‐control layer, and a protective backing. Particular emphasis is placed on sustainable polymers, including cellulose derivatives, alginate, chitosan (CS), polyethylene oxide (PEO), carboxymethyl chitosan (CMCS) and oxidized polysaccharides, together with scalable manufacturing routes such as pressurized gyration (PG). The review also evaluates fluorescein isothiocyanate (FITC)‐dextran and other model macromolecules, highlighting what they prove about physical transport and what they cannot prove about therapeutic bioactivity. Finally, design rules are proposed for next‐generation microneedle‐reservoir patches that integrate biomaterial selection, release control, green manufacturing, and translational requirements.

## Graphical abstract

Hydrogel‐forming microneedle (HFMN)‐reservoir patches provide a modular route for controlled transdermal delivery of hydrophilic biomacromolecules. The platform integrates skin‐barrier penetration, sustainable biopolymers, scalable green manufacturing, hydration‐driven transport, model‐cargo validation, and translational quality requirements. By separating drug storage from the microneedles, this architecture supports higher payload capacity, controlled release, and more practical development of next‐generation biomacromolecule patches.

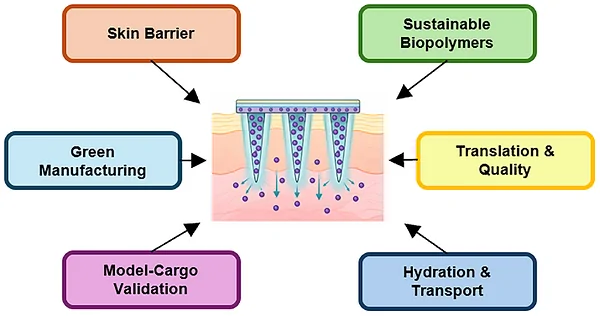

## 1 Introduction

### 1.1 Clinical Need for Transdermal Biomacromolecule Delivery

Hydrophilic biomacromolecules, including insulin, peptide therapeutics, proteins and vaccine antigens, remain difficult to administer without invasive delivery because their molecular size, polarity and structural complexity restrict oral and passive transdermal absorption [1, 2, 3, 4]. Insulin illustrates this challenge directly. It has a molecular mass of approximately 5.8 kDa and poor passive skin permeability, while repeated injections can impose substantial adherence and acceptability burdens during chronic treatment [5, 6]. The relevant delivery challenge is therefore not simply to avoid hypodermic injections, but to provide reproducible dosing while preserving biomacromolecule stability and enabling practical administration. These requirements favor transdermal systems that combine reliable skin access with adequate payload capacity, controlled release, and manufacturable device architecture.

### 1.2 Skin‐Barrier Constraints and the Need for Microneedle‐Mediated Transport

The stratum corneum (SC) is the principal barrier to passive transdermal transport. Although only approximately 10–20 µm thick, its lipid‐rich organization strongly restricts the diffusion and partitioning of hydrophilic molecules and generally favors compounds with molecular masses below approximately 500 Da [7, 8, 9]. Protein‐ and peptide‐sized therapeutics therefore cannot usually achieve clinically useful passive transdermal flux. Chemical permeation enhancers, vesicles and nanocarriers can improve transport for selected molecules, but remain insufficient for reproducible delivery of many hydrophilic biomacromolecules without physical disruption of the barrier [10, 11, 12, 13]. Microneedles provide such access while limiting penetration depth relative to conventional hypodermic needles, establishing a basis for reservoir‐coupled systems in which skin penetration, drug storage, and release control can be engineered as separate functions [14, 15, 16], as illustrated in Figure 1.

**FIGURE 1:**
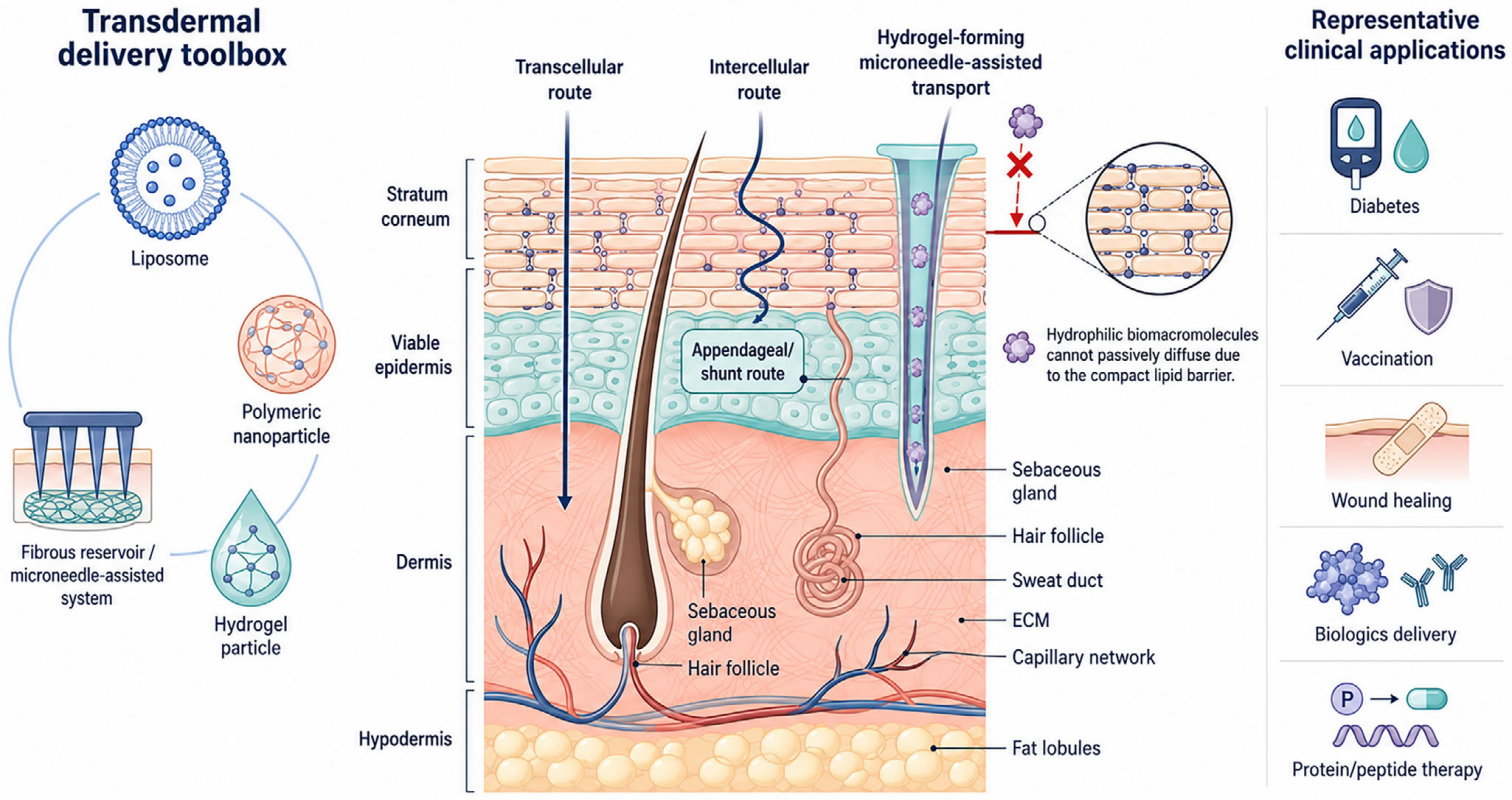
Schematic overview of skin‐barrier limitations and carrier‐ or microneedle‐assisted transdermal strategies for hydrophilic biomacromolecule delivery and clinical translation. Adapted from Innovative transdermal drug delivery systems: benefits, challenges, and emerging applications under the terms of the Creative Commons Attribution 4.0 License [9].

### 1.3 Why Microneedles Changed the Field but Have Not Solved Translation

Microneedles changed the field by physically bypassing the SC rather than relying on passive partitioning. They can reduce insertion depth compared with hypodermic needles and improve patient acceptability, as shown conceptually in Figure 2 [14, 15, 16, 17]. Nevertheless, penetration alone is not sufficient for biomacromolecule therapy. The remaining translation barriers are loading capacity, dose uniformity, sustained flux, reservoir sealing, microbiological quality, storage stability, mechanical reproducibility, and regulatory classification as drug‐device combination products [10, 18, 19]. A key message is that microneedle translation is limited by system integration rather than by skin puncture alone. If the patch cannot reproducibly load a biologic, preserve it during manufacture, release it over the intended time window, and meet the required microbiological and stability specifications during storage, then successful insertion has limited translational value [1, 10, 18].

**FIGURE 2:**
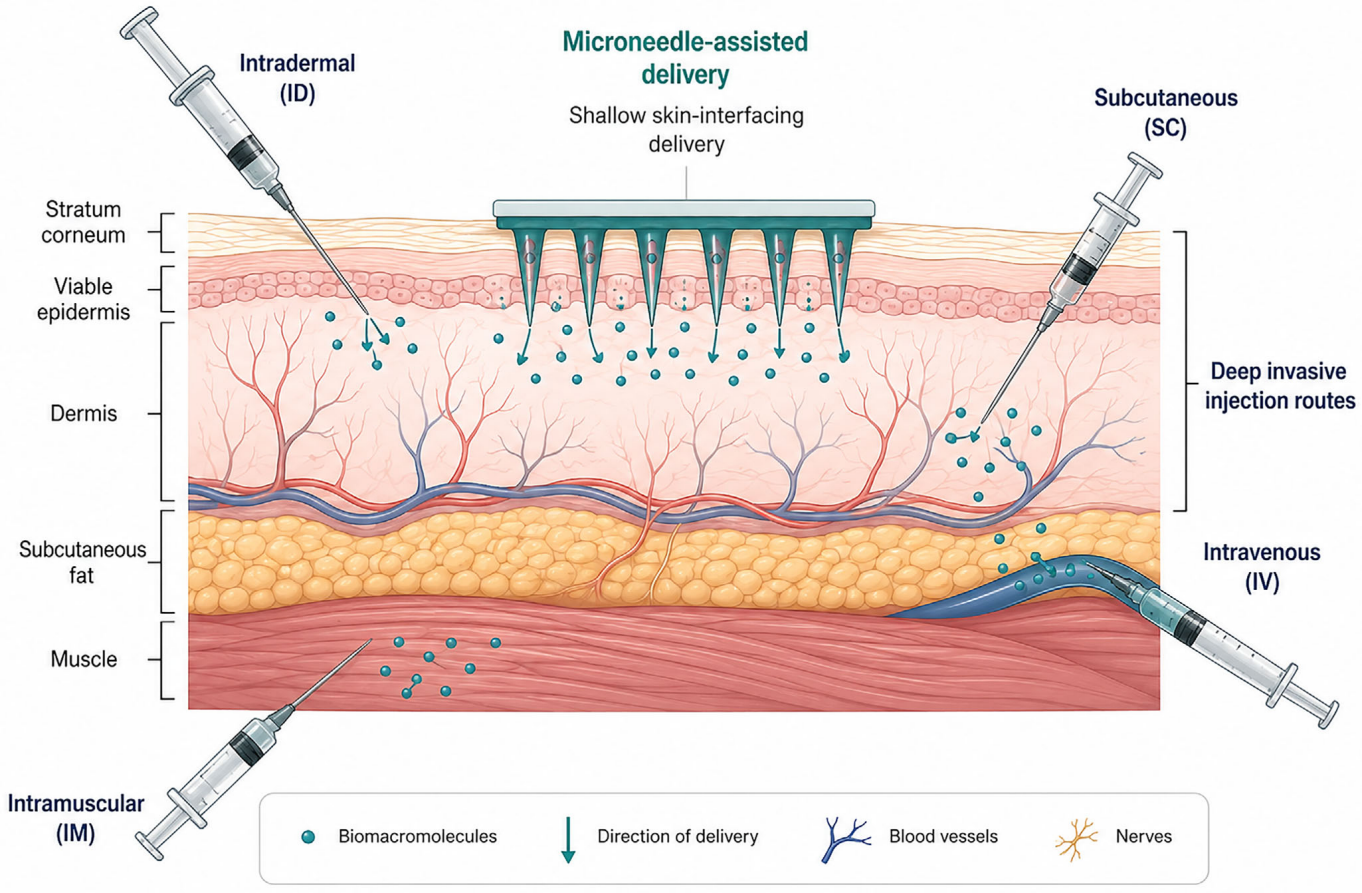
Schematic comparison of shallow microneedle‐assisted biomacromolecule delivery with conventional intradermal, subcutaneous, intravenous and intramuscular injection routes. Adapted from Polymeric microneedle advancements in macromolecule drug delivery: current trends, challenges, and future perspectives with permission from Springer Nature. Copyright 2025, The Author(s), under exclusive license to Springer‐Verlag GmbH Germany, part of Springer Nature [4].

Specific examples illustrate why microneedle translation is limited by integrated device performance rather than skin puncture alone. Solid microneedles can validate the “poke” step, but they still require a separate formulation or patch to deliver the drug after microchannel formation, whereas coated microneedles simplify dosing but remain restricted by surface loading, while hollow microneedles can decouple the liquid reservoir from the needle body but introduce risks of bore blockage, tissue compression, and pumping complexity [4, 19, 20]. Hydrogel forming microneedle (HFMN)‐reservoir systems provide a more integrated solution because the microneedles swell to form aqueous conduits while an external reservoir controls dose loading and release, as reported by reservoir‐HFMN studies delivering milligram‐scale payloads such as ibuprofen sodium and ovalbumin over 24 h [21, 22]. These examples show that the translational question is not simply whether microneedles enter skin, but whether the complete system can load, preserve, release, and deliver biomacromolecules reproducibly.

Although previous studies have provided valuable accounts of microneedle classifications, HFMN materials, transdermal transport mechanisms, and emerging clinical opportunities, the field remains fragmented around the microneedle itself rather than the complete delivery architecture [1, 4, 23, 24]. Similarly, recent studies show that microneedles are evolving from passive skin‐poration structures into integrated platforms that combine delivery, sensing, feedback, and responsive therapy within one skin‐interfacing device [25]. This review addresses that gap by repositioning HFMN as one component of a multilayer microneedle‐reservoir system designed specifically for hydrophilic biomacromolecules. The central argument is that successful translation will depend not only on skin penetration, but on the coordinated design of the hydrogel conduit, drug reservoir, diffusion‐control layer, protective backing, model‐cargo validation strategy, scalable manufacturing route, and regulatory pathway. By integrating sustainable polymer selection, fiber‐forming technologies such as pressurized gyration (PG), fluorescein isothiocyanate (FITC)‐dextran interpretation, quantitative characterization standards, and translational design rules, this review moves beyond conventional types of microneedles narratives and establishes a device‐level framework for engineering reproducible, scalable, and sustainable transdermal biomacromolecule delivery platforms. Table 1 summarizes the principal quantitative constraints that define the transdermal delivery challenge for hydrophilic biomacromolecules.

**TABLE 1:** Quantitative constraints defining the transdermal biomacromolecule‐delivery problem.

Constraint	Representative value	Implication	Refs.
SC thickness	10–20 µm	Small physical thickness but dominant diffusion barrier	[7, 9]
Passive molecular‐weight boundary	<500 Da	Excludes most peptides, proteins, and dextran	[8, 9]
Insulin size and hydrophilicity	∼5.8 kDa; logP ‐1.61	Poor passive skin partitioning and diffusion	[5, 6]
Injectable‐insulin adherence	<50%	Supports need for less burdensome delivery	[6]
Needle phobia in diabetic patients	∼35%	Psychological barrier to repeated therapy	[6]
Passive patch dose window	<10 mg/day for many conventional systems	Insufficient for many hydrophilic biologics without active enhancement	[1, 9]

## 2 From Microneedles to Microneedle‐Reservoir Systems

### 2.1 Drug‐in‐Needle Systems

Previous studies have classified microneedles as solid, coated, hollow, dissolving, or hydrogel‐forming. However, for biomacromolecule delivery, this conventional material‐geometry classification is less useful than an architectural classification based on where the payload is stored and how it reaches the skin. In a drug‐in‐needle system, the therapeutic is either deposited on the microneedle surface, as in coated microneedles, or incorporated within the dissolving microneedle matrix. These systems are attractive for vaccines, antigens, and highly potent drugs because insertion and release occur from the same microstructure. However, this architecture creates a volumetric and mechanical ceiling, as coated microneedles are limited by coating surface area, while dissolving microneedles must balance drug loading against needle strength, dissolution behavior, and polymer deposition in the skin [4, 26, 27]. This architectural framework separates drug‐in‐needle systems, hollow needle‐as‐channel systems, hydrogel‐forming needle‐as‐conduit systems and reservoir‐controlled systems, allowing the limitations of coated, dissolving and hollow microneedles to be compared directly against the requirements of sustained biomacromolecule delivery [21, 22, 28].

### 2.2 Needle‐as‐Conduit Systems

A needle‐as‐conduit system stores the therapeutic outside the microneedle and uses the array primarily as a transport interface between an external reservoir and viable skin. HFMNs perform this function after insertion by forming a hydrated pathway through which hydrophilic molecules can pass from the reservoir into the skin [21, 23, 29], as illustrated in Figure 3. In contrast to drug‐in‐needle systems, dose capacity is therefore governed mainly by the reservoir rather than by the finite volume of the microneedle projections. For biomacromolecule delivery, the principal device‐level requirements are reliable reservoir contact, sufficient hydration, and maintenance of a concentration gradient across the HFMN interface [22, 30, 31].

**FIGURE 3:**
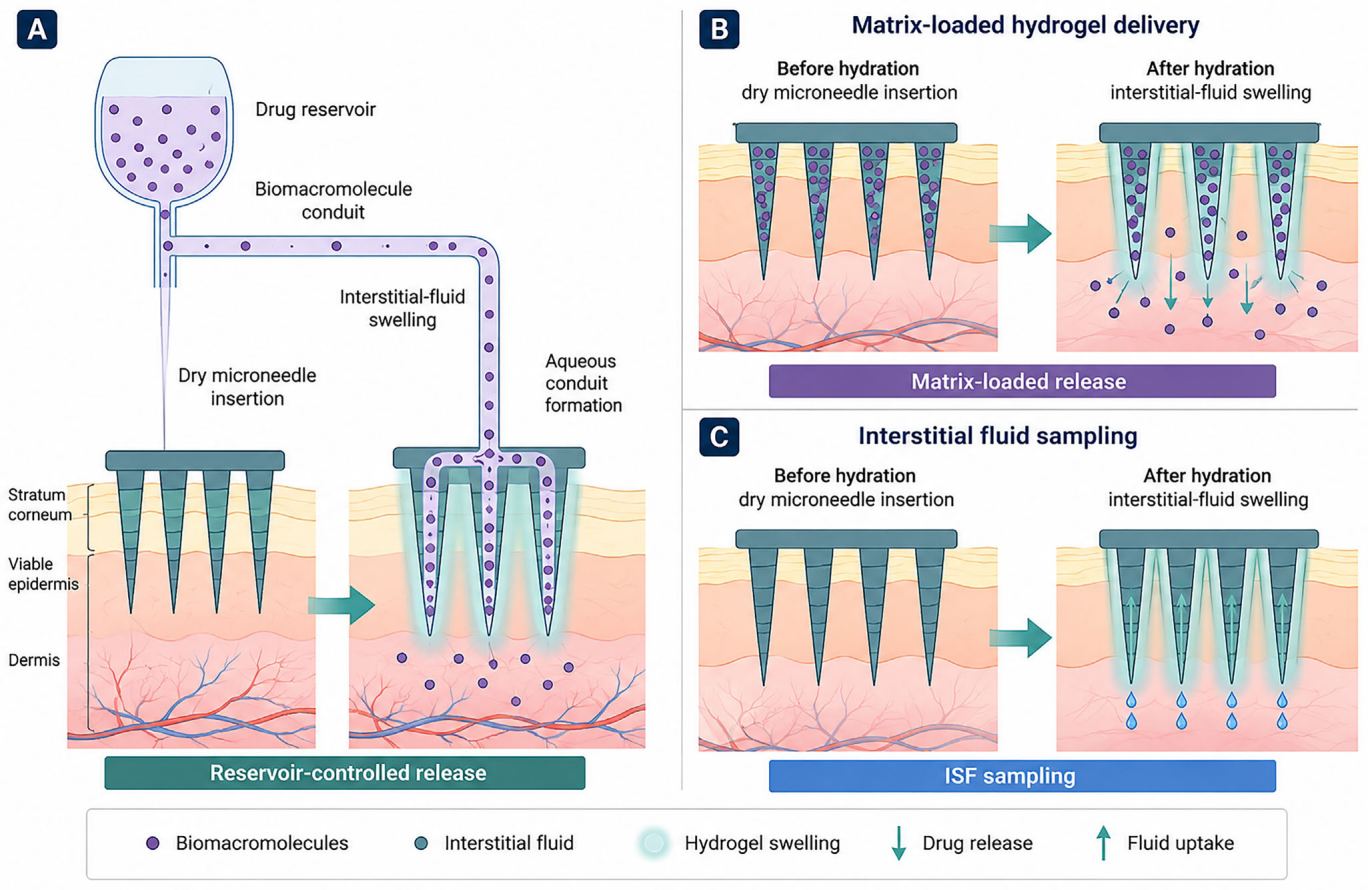
HFMN mechanisms for reservoir‐controlled biomacromolecule delivery, matrix‐loaded release and interstitial fluid sampling after hydration‐mediated swelling. Adapted from Hydrogel‐forming microneedles: current advancements and future trends under the terms of the Creative Commons Attribution 4.0 License [23].

### 2.3 Reservoir‐Controlled Systems

A reservoir‐controlled microneedle system is a multilayer architecture in which the drug is stored outside the microneedle array. Reservoir formats include lyophilized wafer, compressed tablet, liquid compartment, hydrogel depot, fibrous mat, or multilayer polymer patch [21, 22, 28, 32, 33]. Separating the reservoir from the microneedle allows drug loading, reservoir hydration, diffusion resistance, environmental protection, and mechanical insertion to be controlled through different components of the device. Reservoir‐HFMN systems have delivered milligram‐scale payloads such as ibuprofen sodium and ovalbumin over 24 h, whereas coated and dissolving drug‐in‐needle systems are more constrained by surface area or microneedle matrix volume [21, 22].

The reservoir concept also changes how release data should be interpreted. A dissolving microneedle may show rapid local release from the inserted tip, whereas a reservoir‐HFMN system is governed by water uptake, reservoir dissolution, diffusion across the hydrogel conduit, and permeation into skin. Consequently, identical cumulative‐release percentages can reflect very different mechanisms if the donor dose, reservoir geometry, patch area, receptor medium, and skin thickness differ [21, 30, 31, 34].

### 2.4 Why Reservoir Architecture Matters for Biomacromolecules

The value of an external reservoir becomes most apparent when the required dose or delivery duration cannot be accommodated within the microneedle itself. This does not make reservoir‐HFMNs universally preferable. Coated microneedles can be appropriate for highly potent molecules requiring small doses and rapid release, dissolving microneedles provide a comparatively simple single‐use format for vaccines and peptides, and hollow microneedles allow direct liquid administration when pressure‐controlled delivery is acceptable [4, 19, 20, 27, 28]. Reservoir‐HFMNs become particularly relevant when higher loading capacity or sustained delivery of hydrophilic macromolecules is required, but this is accompanied by hydration‐dependent lag time, reservoir coupling, sealing, and greater assembly complexity [21, 22, 30, 31]. Table 2 therefore compares these architectures according to their principal advantages, limitations, quantitative performance, and appropriate therapeutic context.

**TABLE 2:** Suitability comparison of microneedle architectures for biomacromolecule delivery.

Architecture	Principal advantage	Principal limitation	Appropriate clinical scenario	Representative quantitative evidence	Refs.
Coated drug‐in‐needle	Rapid release and relatively simple dose placement	Surface‐area‐limited loading and potential loss of tip sharpness with excessive coating	Vaccines, antigens and highly potent therapeutics requiring small doses and rapid release	<50–100 µg payload in some coated systems; 0.3 mg peptide payload reported for 3M sMTS	[4, 19]
Dissolving drug‐in‐needle	Payload incorporated throughout a water‐soluble microneedle matrix with no rigid sharps remaining after use	Mechanical‐payload trade‐off, polymer deposition in skin and release coupled to matrix dissolution	Vaccination, potent peptides and single‐use delivery where moderate payload capacity is sufficient	58.5% active delivery of IgG‐HRP in one active dissolving system; LNG release of 60%–90% over several days	[35, 36]
Hollow needle‐as‐channel	Liquid dose and infusion rate can be controlled independently of solid microneedle loading capacity	Bore clogging, leakage, and requirement for pressure or pumping control	Rapid liquid delivery or controlled infusion where an external delivery mechanism is acceptable	Maximum insulin flow rate of ∼45 µL min^−1^ achieved using an SU‐8 hollow‐microneedle array integrated with a Nafion‐actuated micropump	[2]
Reservoir‐HFMN	Drug loading is decoupled from microneedle volume, and sustained delivery can be achieved while retaining the crosslinked array for removal	Hydration‐dependent lag time, reservoir‐to‐array coupling, sealing, dose uniformity, and greater assembly complexity	Sustained or higher‐capacity delivery of hydrophilic macromolecules where longer application periods are acceptable	44 mg ibuprofen sodium and ∼1.2 mg ovalbumin delivered over 24 h in reservoir‐HFMN studies	[21]

The quantitative values presented in Table 2 are drawn from independent studies conducted under different experimental conditions and are included to illustrate representative performance ranges rather than enable direct comparison between microneedle platforms.

### 2.5 Evidence Landscape of Microneedle Systems for Macromolecular Delivery

Available evidence does not support a single microneedle architecture for all biomacromolecular therapies. Coated microneedles provide rapid delivery and have demonstrated clinical potential for potent peptide therapeutics, but their surface‐area‐limited loading capacity becomes restrictive as dose requirements increase [4, 19]. Dissolving microneedles accommodate greater payloads within the polymer matrix and are particularly attractive for vaccines and self‐administered peptide delivery, although increasing drug loading may compromise mechanical integrity and repeated use results in deposition of the dissolving polymer matrix within viable skin [2, 26, 37, 38, 39, 40]. Hollow microneedles avoid a solid‐matrix loading constraint and can provide rapid insulin absorption or direct administration of macromolecular formulations, but their performance depends on maintaining an unobstructed lumen and controlled liquid delivery [20, 41, 42, 43, 44]. Reservoir‐HFMNs decouple drug storage from microneedle volume and have enabled milligram‐scale and sustained delivery, including protein‐sized payloads, but require hydration before steady transport is established and introduce additional requirements for reservoir attachment, sealing and device assembly [21, 22, 28].

Clinical suitability therefore depends on the intended dose, release duration, cargo stability and administration setting. Coated systems are well matched to low‐dose therapeutics requiring rapid release. Dissolving systems are attractive when a simple disposable format and complete matrix dissolution are desirable. Hollow systems are suited to controlled liquid administration when an external pressure source or applicator can be accommodated. Reservoir‐HFMNs offer a stronger rationale when sustained delivery or greater payload capacity is required, provided that hydration lag time, prolonged skin contact, and increased manufacturing complexity are acceptable. The choice of architecture should therefore follow the target product profile rather than an assumption that increasing structural complexity necessarily improves therapeutic performance.

### 2.6 Representative Microneedle Platforms for Hydrophilic Macromolecule Delivery

The delivery of hydrophilic macromolecules, including peptides, proteins, vaccines and nucleic‐acid therapeutics, remains limited by the lipid‐rich SC barrier, which restricts passive transport of large polar molecules [4, 45]. Microneedle platforms address this limitation by creating transient aqueous microchannels, but their translational suitability depends strongly on whether the payload is stored within the needle, delivered through a hollow channel, or released from an external reservoir [4, 20, 23]. Table 3 summarizes representative microneedle systems for macromolecule and model‐macromolecule delivery, together with relevant long‐acting comparator systems that inform reservoir design.

**TABLE 3:** Representative microneedle platforms for hydrophilic macromolecule delivery.

Payload/model molecule	Microneedle type	Material or reservoir system	Key quantitative evidence	Main limitation	Refs.
Insulin	Dissolving	Starch/gelatin dissolving matrix	>90% insulin retained after 1 month at 37 °C, with ∼92% relative pharmacological availability reported after delivery.	Polymer deposition and mechanical‐payload trade‐off remain concerns for repeated use.	[38]
Glucagon	Glucose‐responsive microneedle patch	Shrinking‐fabricated polymeric matrix	Shrinking fabrication enabled formation of miniaturized glucose‐responsive glucagon microneedles while reducing cargo loss during processing.	Responsive hormone delivery still requires robust glucose‐trigger calibration and long‐term stability validation.	[46]
Synthetic mRNA	Hollow	Liquid infusion through hollow microneedles	Intradermal mRNA delivery enabled rapid production of exogenous proteins in skin.	Requires reliable fluidic actuation and may need active pumping.	[44]
Diverse actives, including macromolecules	Ice microneedles	Frozen water or biopolymer‐based microneedle tips	Ice microneedles enabled rapid melting after insertion and residue‐minimized delivery of diverse active agents.	Storage, handling, and cold‐chain practicality remain important translational constraints.	[47]
FITC‐dextran, 4 kDa	Dissolving	PLGA/PVA composite microneedles	Enhanced *ex vivo* macromolecule delivery compared with passive patches.	Higher loading can increase brittleness and weaken insertion.	[48]
Parathyroid hormone, PTH 1–34	Coated	Coated titanium Macro flux array	Reported faster systemic exposure than subcutaneous injection in reviewed clinical evidence.	Surface‐area‐limited loading restricts high‐dose use.	[4, 45]
IgG‐HRP	Active hybrid	Magnesium‐polymeric active microneedle array	58.5% active delivery efficiency reported within 15 min.	Hydrogen‐generation mechanism adds formulation and regulatory complexity.	[36]
Bevacizumab	Reservoir‐HFMN	Hydrogel‐forming combination patch	Enabled delivery of a monoclonal antibody to systemic and lymphatic compartments.	Reservoir‐needle integration and dose recovery require careful control.	[28]
Ovalbumin	Reservoir‐HFMN	Gantrez‐based HFMNs with lyophilized wafer reservoir	1.24 mg delivered over 24 h with 49% delivery efficiency.	Continuous skin contact and sufficient hydration are required.	[21]
Octreotide acetate	Hollow	LCD‐vat‐polymerized hollow microneedles	Insertion force of 570 mN per microneedle reported for high‐precision 3D‐printed structures.	High aspect‐ratio hollow tips remain vulnerable to breakage and clogging.	[49]
DNA vaccine	Coated	pH‐sensitive charge‐reversal copolymer coating	Improved antigen‐presenting‐cell homing and immune responses.	Coating variability can compromise dose uniformity.	[50]
STAT3 siRNA	Dissolving	Chitosan/biodegradable microneedle matrix	Intradermal delivery investigated for melanoma immunotherapy applications.	Nucleic‐acid stability during processing and storage remains a key barrier.	[51]
Cefazolin	Reservoir‐HFMN	Dry reservoir‐HFMN composite	∼20 mg cumulative permeation reported in porcine skin.	Release remains dependent on hydration and concentration‐gradient maintenance.	[33]
FITC‐BSA and insulin	Dissolving	Pullulan‐based microneedle array	Pullulan DMNs preserved biomolecule stability and enabled ex vivo porcine‐skin permeation.	Rapid dissolution limits sustained delivery unless combined with a reservoir.	[52]
Etravirine	Dissolving bilayer	Bilayer polymeric microneedle array	Long‐acting delivery achieved in a polymeric microneedle format.	Mechanical‐payload trade‐offs increase with high drug loading.	[53]
Antimicrobial and immunomodulatory wound therapy	Functional microneedle patch	Nanoparticle‐integrated polymeric microneedle matrix	Local microneedle delivery combined antimicrobial and immunomodulatory functions to promote infected‐wound healing.	Translation requires infection‐specific safety, dose control, and repeat‐application evidence.	[54]

The evidence landscape shows a clear shift from drug‐in‐needle concepts toward reservoir‐coupled or long‐acting systems for macromolecular delivery. Coated and dissolving microneedles remain attractive for vaccines, peptides and potent compounds, but they are constrained by limited surface or matrix volume and by the mechanical‐payload trade‐off that emerges when drug loading increases [4, 45, 48]. Hollow microneedles can deliver larger liquid volumes, including nucleic‐acid formulations, but clogging, pumping requirements, and fabrication complexity limit their routine translation [41, 44]. Reservoir‐coupled HFMNs are therefore particularly important for hydrophilic biomacromolecules because the swollen hydrogel interface controls transport while the external reservoir determines dose capacity [21, 22, 33].

## 3 Hydrogel‐Forming Microneedles (HFMNs)

### 3.1 Swelling, Insertion and Aqueous Conduit Formation

The dry‐to‐hydrated transition is the defining scientific problem in HFMN. Before insertion, the hydrogel network is dehydrated and glassy, allowing the needle to resist buckling and penetrate the SC. After insertion, water from interstitial fluid diffuses into the polymer network, solvates hydrophilic chains, and relaxes the matrix from a rigid xerogel into a swollen hydrogel [23, 29, 55, 56, 57]. Swelling increases the free volume and mesh size available for solute transport, but it also reduces stiffness and can promote deformation if the network is insufficiently crosslinked. Thus, a highly crosslinked network may insert well but restrict macromolecular diffusion, whereas a weakly crosslinked network may swell rapidly but deform, collapse, or detach during wear. The design challenge is therefore to balance dry mechanical strength with hydrated permeability, so that the microneedle first functions as a penetration tool and then as a stable aqueous conduit [24, 58, 59].

### 3.2 Mechanical Requirements

Insertion performance is governed by the interaction between microneedle geometry, material stiffness, and skin deformation. Needle height determines the maximum possible penetration depth, base width affects load‐bearing capacity, tip radius controls the stress concentration at the skin surface, and aspect ratio influences the tendency to buckle or fracture during compression [60, 61]. In simplified mechanical terms, the applied insertion force must exceed the force required to pierce the SC but remain below the fracture force of the needle. Height reduction after compression is therefore not a minor characterization value; rather, it indicates whether the needle plastically deforms before insertion, while fracture force defines the safety margin against tip breakage in skin [24, 61]. Values of ∼58–100 mN per needle are often cited as relevant for skin insertion, but whole‐array force, force per needle, and actual insertion depth have to be reported separately because dense arrays can distribute the applied load and generate a bed‐of‐nails effect [60, 61, 62, 63]. Evidence that array pitch and skin deformation are not secondary design details, but central determinants of whether microneedles insert effectively is shown in Figure 4.

**FIGURE 4:**
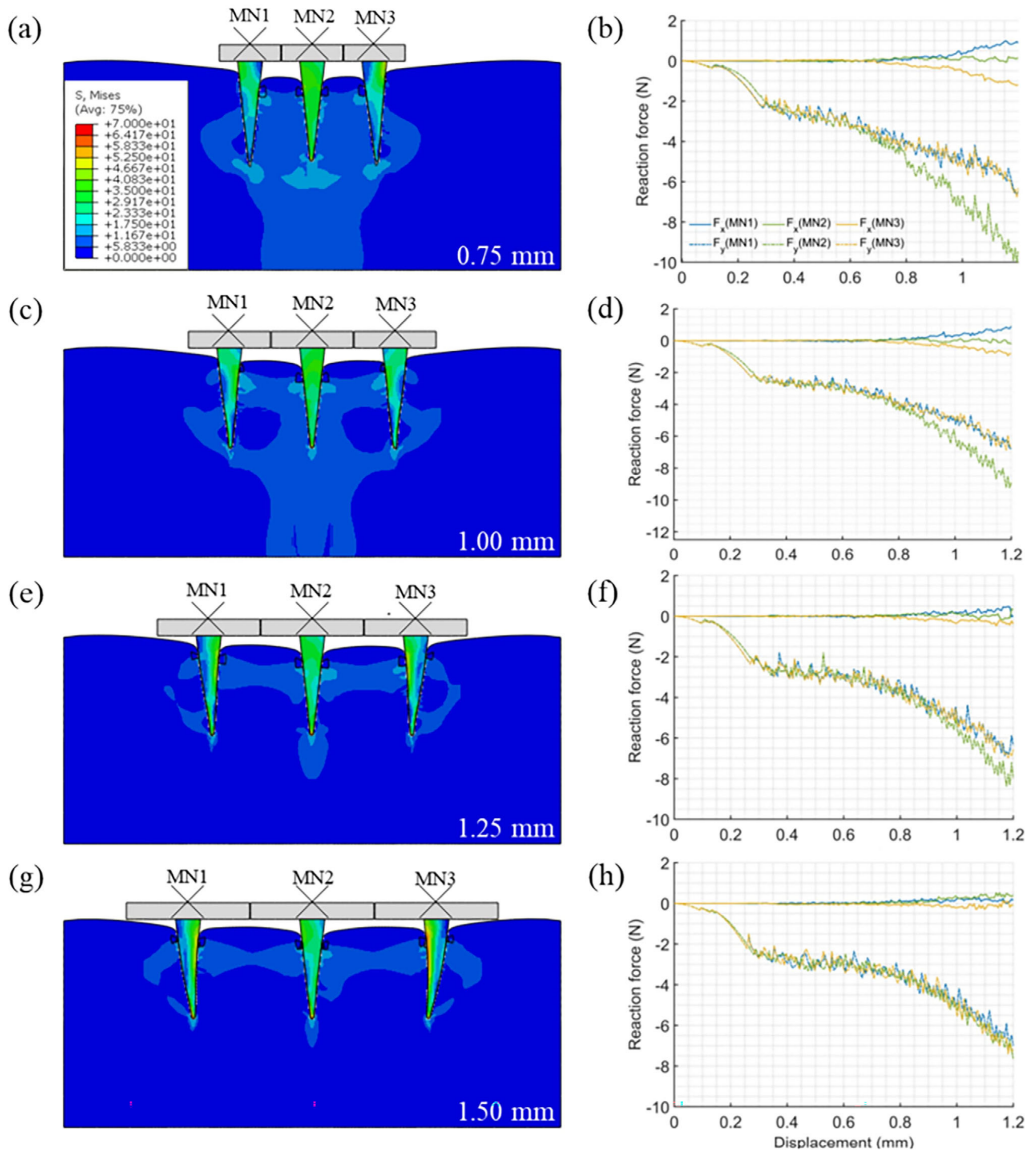
Influence of microneedle interspacing on insertion mechanics. Finite‐element stress distributions and reaction‐force profiles show how array pitch influences penetration and the bed‐of‐nails effect. From Finite element analysis of polymeric microneedle insertion into skin under the terms of the Creative Commons Attribution 4.0 License [60].

### 3.3 Synthetic versus Natural HFMNs

Synthetic and semi‐synthetic HFMNs set the performance benchmarks that natural systems must match. Gantrez/PEG (Polyethylene Glycol) systems which have shown high swelling capacity, robust insertion, and reservoir compatibility, with reported mass increases >1000% and reaching ∼1700%–2200% depending on formulation and test duration [21, 56]. Polyvinylpyrrolidone (PVP)/ Polyvinyl alcohol (PVA) and PEG‐based systems provide further control over swelling, mechanical behavior, and release, although thermal or chemical crosslinking conditions may not always be ideal for sensitive biomacromolecules [64, 65, 66, 67].

Natural and semi‐natural systems, including chitosan (CS), carboxymethyl chitosan (CMCS), hyaluronic acid (HA), pullulan and oxidized polysaccharides, are attractive because they offer water compatibility, functional groups for dynamic crosslinking and potential biological advantages. However, their value must be demonstrated quantitatively. For example, CMCS‐SFP (Silk Fibroin Peptide) /OPL (Oxidized Pullulan) hydrogel microneedles have been reported with a fracture force of 88 mN per needle, providing a useful mechanical benchmark for polysaccharide‐based systems [68], with their array morphology and structural fidelity shown in Figure 5. Therefore, natural hydrogel microneedles should be framed as performance‐competitive alternatives to synthetic systems, not as automatically superior materials.

**FIGURE 5:**
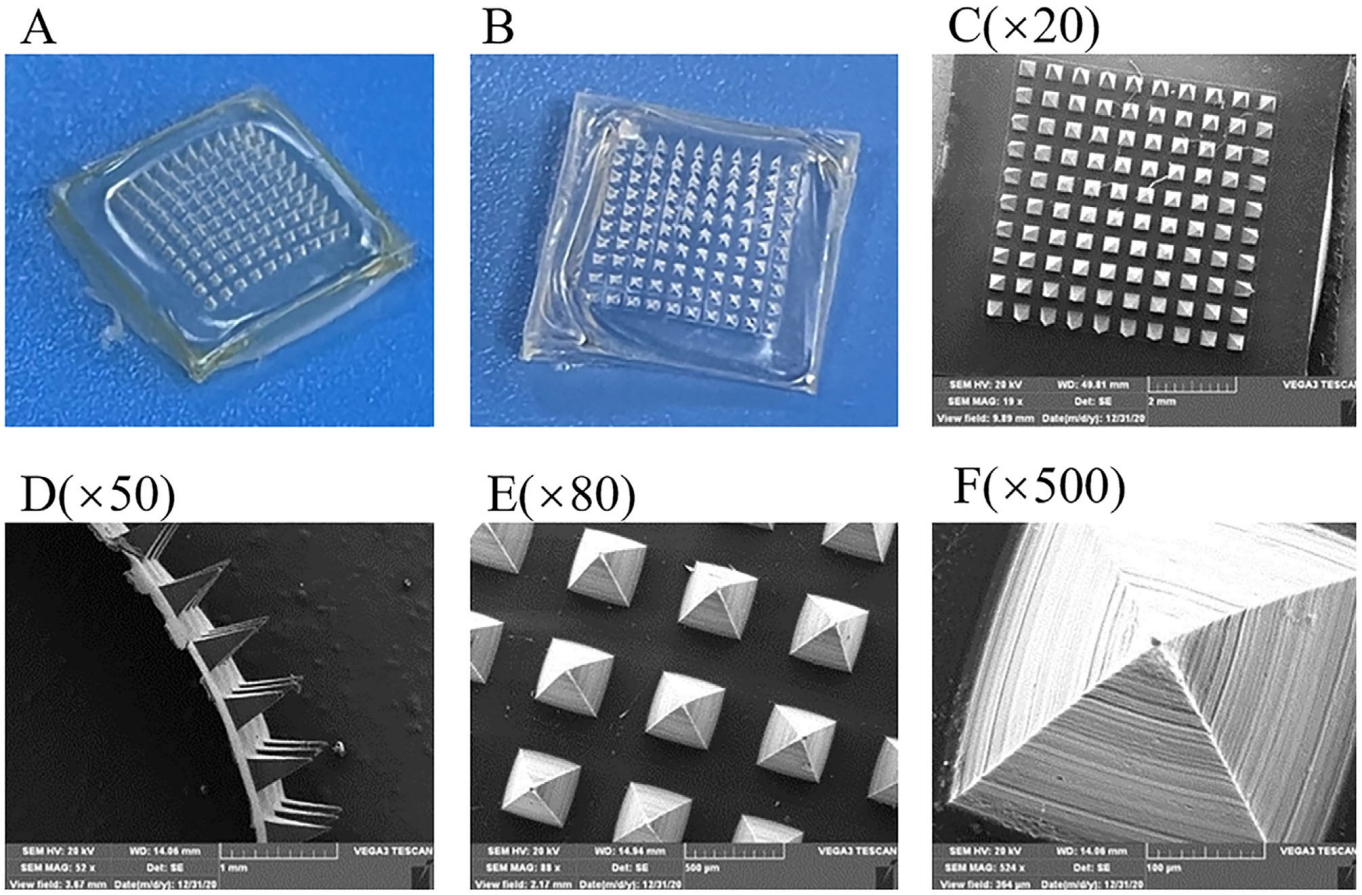
CMCS/OPL hydrogel microneedle morphology. Optical and scanning electron microscopy images show how CMCS‐SFP/OPL composition affects array shape, geometry, and structural fidelity. From Hydrogel‐based microneedles of chitosan derivatives for drug delivery (Copyright 2022 Elsevier B.V.) [68].

### 3.4 Sustainability Limits of Current Hydrogel Microneedles

Gantrez, PEG, PVA, and PVP systems have strong performance records, but their environmental profile depends on polymer source, crosslinking chemistry, drying energy, microbiological‐control requirements, compatibility with sterilization where required, and end‐of‐life handling [21, 24, 61]. Similarly, a CS or polysaccharide microneedle is not automatically sustainable if it requires toxic crosslinkers, aggressive solvents, long energy‐intensive drying, or poor shelf stability [69, 70].

A further limitation is that many HFMN studies evaluate freshly prepared arrays under ideal laboratory conditions, whereas translational devices must retain their performance after manufacture, microbiological control, packaging and storage. The appropriate microbiological specification may involve a sterile or low‐bioburden product depending on the product‐specific infection‐risk assessment [71]. Dry hydrogel networks can absorb moisture during storage, lose tip sharpness, change crosslink density, or alter payload distribution. These issues are rarely addressed in early proof‐of‐concept work [18, 61, 72]. Sustainability claims must therefore be considered alongside shelf‐life and quality‐control evidence.

### 3.5 Hydrogel Network Chemistry and Swelling‐Transport Design

HFMNs are governed by a deliberately balanced network design like the array must be sufficiently stiff in the dry xerogel state to pierce the SC, yet sufficiently hydrated and permeable after insertion to transport macromolecules [23, 29]. This dry‐to‐swollen transition is controlled by crosslinking density, polymer chain mobility, free volume, and effective mesh size, which together determine swelling ratio, hydrated stiffness, and solute diffusion [56]. For biomacromolecules, the hydrogel network should therefore be considered a transport‐selective interface rather than merely a swelling material. Recent work on swelling‐shrinkable hydrogels reinforces that swelling should be treated as a programmable mechanical response rather than only a water‐uptake value, because controlled swelling, anti‐swelling, or shrinkage can influence shape retention, wound closure, and drug‐release behavior [73]. Fibrous hydrogel architectures are also relevant to microneedle‐reservoir design because internal fiber networks can improve toughness, directional mechanics, and transport control in soft biomedical interfaces [74].

Covalently crosslinked systems remain important because they maintain structural integrity during prolonged skin residence. Gantrez S‐97/PEG networks use thermal esterification to produce robust hydrogel‐forming arrays, and Fourier Transform Infrared Spectroscopy (FTIR)‐derived crosslinking factors have been used to relate network formation to mechanical strength and swelling behavior [29, 56]. Super‐swelling variants can show very high mass increases by incorporating sodium carbonate, which ionizes carboxyl groups and expands the network through electrostatic repulsion [56]. PVA/PVP and PEG‐based networks similarly allow tuning of swelling and permeation, but thermal treatment, residual crosslinkers, and processing conditions must be compatible with sensitive biomacromolecules [65, 67].

Natural and semi‐natural polymers offer water compatibility and functional chemistry, but they generally require modification or crosslinking to prevent premature dissolution. Methacrylate HA systems can be UV‐crosslinked to control extraction and delivery, and the curing time strongly influences swelling and mechanical robustness [75]. CS‐based systems can use Schiff‐base chemistry, genipin crosslinking, or semi‐dissolving dual‐domain structures to combine early hydration with sustained release [68, 70, 76]. OPL, pectin/Gantrez and related polysaccharide networks add aldehyde, carboxylate or hydroxyl functionalities that support dynamic interactions and high‐water uptake [68, 77].

The transport of FITC‐dextran, insulin, bovine serum albumin (BSA), or albumin‐like payloads through swollen networks depends on the ratio between the solute hydrodynamic radius and the effective hydrogel mesh size [23, 56, 78]. When the mesh size approaches or falls below the solute dimensions, diffusion becomes sterically hindered, and release may be governed by anomalous transport involving both water diffusion and polymer relaxation [56]. This explains why swelling ratio alone is an insufficient criterion, as a highly swollen but mechanically weak hydrogel may lose dimensional stability, while an overly crosslinked network may resist deformation but restrict macromolecular flux.

A major unresolved challenge is hydrated weakness. Excessive swelling can reduce modulus, cause lateral deformation, and increase the risk of needle bending or fracture during wear and removal [23]. Therefore, the hydrogel network must maintain a mesh size large enough for biomacromolecule diffusion while preserving a hydrated modulus sufficient for stable residence and intact removal [56, 67, 79].

### 3.6 Microneedle Geometry, Applicators and Insertion Reliability

The clinical translation of microneedle‐reservoir patches depends on reproducible formation of the skin‐needle interface, which is governed by the coupled effects of microneedle geometry, material stiffness, and skin biomechanics [60, 80]. Needle height determines the maximum possible insertion depth, but actual penetration is reduced by the viscoelastic deformation of skin during application [80]. In comparative penetration studies, 500 µm needles were often less reliable in porcine and rat skin because the baseplate contacted the skin before the needle tips were fully seated, whereas 1000 µm needles allowed clearer separation between tip insertion and baseplate contact [80]. Therefore, needle height should be selected according to target layer, skin deformation, and patient comfort rather than by nominal length alone [80, 81].

Tip radius and base width control the balance between insertion force and fracture resistance. Sharper tips concentrate stress more effectively at the SC; thereby, reducing the tip radius from 37 µm to 5 µm has been associated with a reduction in insertion force from 167 mN to 20 mN per needle [23]. However, sharp and high‐aspect‐ratio structures are also more susceptible to buckling, particularly when insertion is misaligned or when lateral skin forces are applied during wear [60, 82]. Compression testing of individual polyglycolic acid microneedles reported a mean buckling load of 210 ± 10 mN per needle, illustrating the narrow mechanical window between effective insertion and failure [63]. For HFMNs, crosslinked Poly (ethylene glycol) diacrylate (PEGDA) and Poly (methyl vinyl ether) (PMVE)/ Maleic acid (MA)‐pectin systems show that polymer composition can tune fracture resistance, height reduction, and swelling, but these parameters must be interpreted together [67, 77].

Array density further complicates insertion because the applied force is distributed over many tips. The bed‐of‐nails effect can reduce penetration efficiency in high‐density arrays, with lower‐density configurations often producing deeper insertion under the same total force [60, 83]. Finite‐element analyses suggest that increasing pitch reduces constraint of the skin between adjacent needles, allowing more local deformation and more reliable penetration [60]. Supporting posts and engineered baseplate structures can also improve insertion probability by locally stretching skin and reducing premature bottoming‐out of the array.

Manual thumb application remains a major source of variability because patients cannot reproducibly control angle, speed, and force. Spring‐loaded or impact‐driven applicators can deliver a defined force and velocity, improving insertion consistency across different users and skin sites [18, 81, 84]. Feedback mechanisms, including audible or tactile confirmation of insertion, can further improve dose confidence by indicating that the array has been applied correctly [18, 81]. Insertion should therefore be validated using a hierarchy of methods, including ex vivo porcine or human skin, dye staining, optical coherence tomography and post‐application recovery studies [21, 29, 60, 61]. Therefore, reliable microneedle‐reservoir patches should combine sharp tips, sufficient base width, moderate array density, and applicator‐controlled insertion so that the fracture force exceeds the dynamic insertion force by a robust safety margin while minimizing pain and avoiding the bed‐of‐nails effect [60, 62].

## 4 Sustainable Polymers for Reservoir‐Based Macromolecular Patches

Sustainable polymer selection for microneedle‐reservoir patches should be discussed as a device‐function problem rather than as a list of materials for a single proposed design. In an integrated reservoir patch, the backing layer, diffusion‐control layer, reservoir, and HFMN interface each impose different requirements. The backing must resist moisture ingress and support directional release, the diffusion layer must regulate hydration and reduce burst release, the reservoir must stabilize and store the macromolecular payload, a and the microneedle interface must insert, swell and remain mechanically intact during wear [21, 22, 23]. Cellulose derivatives, alginate, CS, polyethylene oxide (PEO), CMCS and oxidized polysaccharides are therefore best evaluated according to where they function in the multilayer architecture, how they are processed and whether their performance is supported by quantitative data.

### 4.1 Backing Layers: Hydrophobic Control and Environmental Stability

Reservoir patches require a backing layer that prevents external moisture ingress, protects the payload, and promotes directional release toward the skin. Ethyl cellulose (EC) and cellulose acetate (CA) are attractive because they are established cellulose derivatives with film‐forming and fiber‐forming ability, and EC provides hydrophobic barrier character relevant to outer‐layer design [85, 86, 87]. The ethanol/water route refers to processing polymer solutions using ethanol‐rich mixtures with water as a co‐solvent or non‐solvent modifier, rather than relying entirely on more toxic organic solvent systems. This matters for cellulose‐based drug‐delivery layers because ethanol/water systems can reduce solvent hazards, improve compatibility with pharmaceutical processing, and potentially support more sustainable fabrication where polymer solubility and fiber formation remain adequate [85, 88]. However, some cellulose derivative systems still require solvents such as acetone, Dimethylacetamide (DMAc), or Tetrahydrofuran (THF) to achieve suitable viscosity, evaporation rate, and fiber morphology. Therefore, the sustainability of a cellulose‐based backing layer depends not only on the polymer origin, but on the complete solvent system, process yield, residual solvent control and final barrier performance [69, 89]. The functional integration of this backing with the diffusion‐control layer, wettable reservoir and HFMN array is illustrated in Figure 6.

**FIGURE 6:**
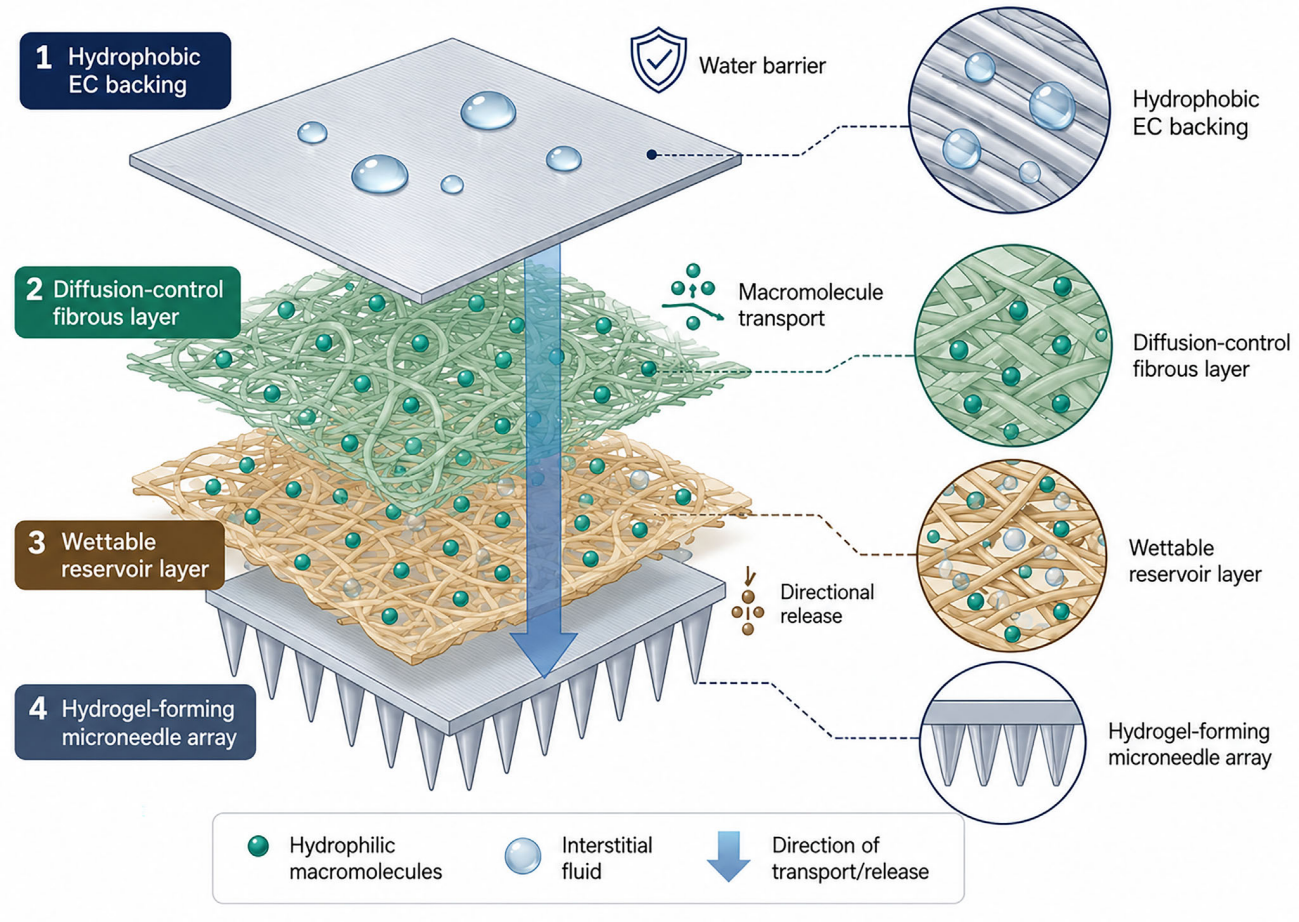
Schematic of the multilayer reservoir‐HFMN patch, comprising a hydrophobic EC backing, a diffusion‐control fibrous layer, a wettable reservoir layer and a HFMN array that together enable directional transport of hydrophilic biomacromolecules toward the skin.

### 4.2 Diffusion‐Control Layers: Alginate and Ionically Crosslinked Hydrogels

The diffusion‐control layer should reduce burst release without blocking macromolecular transport. Calcium‐crosslinked alginate is useful because alginate contains guluronic acid‐rich blocks that can coordinate divalent calcium ions, forming ionic junction zones commonly described as the “egg‐box” structure [90, 91, 92]. In this model, calcium ions bridge adjacent alginate chains, increasing network cohesion and decreasing chain mobility. As crosslink density increases, the hydrogel generally becomes less soluble and more resistant to swelling, while the effective mesh size and water‐filled transport pathways become more restricted. This allows alginate to function as a diffusion‐control layer. Moderate calcium crosslinking can suppress burst release and stabilize the hydrated layer, whereas excessive crosslinking may reduce macromolecular flux by limiting swelling and increasing diffusion resistance [90, 91]. Therefore, alginate should be discussed as a tunable ionic hydrogel rather than simply as a natural polymer, with performance governed by alginate concentration, mannuronic acid/guluronic acid (M/G) ratio, calcium concentration, crosslinking time, pH, ionic strength, and layer thickness.

### 4.3 Reservoir Layers: CS, PEO and Hydrophilic Polymer Networks

Reservoir layers must stabilize hydrophilic cargo, absorb controlled quantities of water, and maintain contact with the hydrogel conduit. CS is attractive because it is cationic, mucoadhesive, and biocompatible, but its processability is strongly governed by protonation of amino groups in acidic media. Acetic acid concentration therefore matters because it controls CS solubility, solution viscosity, chain extension, and spinnability. Too little acid may lead to incomplete dissolution, whereas excessive acid can increase residual acidity, affect biological compatibility, and require additional neutralization after fiber formation [70, 93, 94]. PEO is often introduced as a hydrophilic process aid because it improves chain entanglement and fiber formation, but the chitosan ratio also changes swelling, porosity, and mechanical behavior. CS/PEO reservoirs have produced fiber diameters in the hundreds of nanometers and porosities suitable for hydrated macromolecule‐containing mats, but acetic acid concentration, neutralization conditions, residual solvent, fiber morphology, and wet mechanical stability must be controlled before these systems can be considered translational reservoirs [94, 95]. Nanoparticle–hydrogel hybrid matrices provide a further reservoir‐design strategy because nanoparticles can protect labile cargo, tune release kinetics, and introduce additional responsiveness within a hydrated polymer network [96, 97].

### 4.4 Hydrogel Microneedle Materials: CMCS, OPL and Dynamic Polysaccharide Networks

CMCS, OPL and related dynamic polysaccharide networks are attractive because they can form hydrated structures from water‐compatible chemistries while offering aldehyde, amine and carboxylate interactions that can be tuned for swelling and mechanical performance [68, 76]. These materials provide a credible route away from purely synthetic HFMNs, but their success depends on insertion force, fracture resistance, swelling ratio, protein compatibility and compatibility with the intended microbiological‐control strategy, including sterilization where required, rather than on natural origin alone [68, 71].

### 4.5 Sustainability Evidence

A sustainability claim requires evidence across extraction, solvent selection, fabrication, microbiological control, storage, performance and disposal. Natural origin is insufficient as CS production can be acid/base intensive, cellulose dissolution can require problematic solvents, and glutaraldehyde or other crosslinkers can compromise environmental and biological claims [69, 70, 98]. The more defensible position is to describe specific systems as lower‐solvent, water‐processed, ethanol‐compatible, scalable, or potentially biodegradable only when the data support those claims [18, 99]. Table 4 summarizes the material‐function relationships, representative quantitative evidence, and principal limitations for each layer of a sustainable microneedle‐reservoir patch.

**TABLE 4:** Material‐function matrix for sustainable microneedle‐reservoir patches.

Device layer	Candidate materials	Useful quantitative evidence	Risk or limitation	Refs.
Backing	EC, CA	EC GUHS‐ES yield 24.5 g/h; EC fiber diameters 6–10 µm	Hydrophobicity and stability useful, but the solvent route determines sustainability	[88]
Diffusion‐control layer	Calcium alginate, SA/PEO, CMCS/SA	SA/PEO fibers around 230 nm in an interwoven membrane; alginate mesh and swelling tunable by calcium	Too much crosslinking may suppress macromolecule flux	[90, 91]
Reservoir	CS/PEO, PVP/EC core–sheath, hydrophilic fibrous mats	CS/PEO fiber diameters 313 ± 89 nm to 439 ± 113 nm; reported porosity is 30%–75%	Acetic acid, neutralization, and residual solvent must be controlled	[93, 94, 100]
Hydrogel microneedle interface	CMCS, OPL, HA, pullulan, Gantrez/PEG benchmark systems	CMCS‐SFP/OPL fracture force 88 mN/needle; Gantrez/PEG swelling can exceed 1000%	Natural polymer systems still need dry strength, hydrated integrity, and appropriate microbiological‐quality data	[21, 23, 68, 71]

### 4.6 Adhesion, Sealing and Skin‐Contact Layers

The translational performance of a microneedle‐reservoir patch depends not only on needle geometry or polymer chemistry, but also on the integrity of the skin‐contact and interlayer sealing architecture. In HFMN systems, the reservoir must maintain intimate contact with the microneedle baseplate so that hydration creates a continuous fluidic pathway rather than a discontinuous interface [21, 32]. Poor skin adhesion, reservoir‐edge leakage, or interlayer delamination can convert a designed controlled‐release system into an uncontrolled burst‐release patch [23, 101].

Skin‐contact design must balance adhesion strength, flexibility, and conformability. The baseplate and adhesive must accommodate micro‐scale skin topography so that needles penetrate uniformly across the array [29, 101]. Bullet‐shaped and self‐adherent designs show that microneedle geometry can also contribute to retention through swelling‐mediated mechanical interlocking [102]. Medical adhesive films and occlusive backings such as DuroTak‐coated films or Scotchpak‐type supports have been used in experimental microneedle platforms, but these must be engineered so that they do not block swelling, reservoir hydration, or macromolecule diffusion [21].

Biopolymeric adhesives offer a more sustainable route for prolonged skin contact. CS and its derivatives are attractive because cationic chains can interact electrostatically with negatively charged biological surfaces, while thiolated and catechol‐modified systems can improve wet adhesion through covalent or mussel‐inspired bonding mechanisms [70, 76]. HA, gelatin, alginate, and pectin‐based systems can provide hydration, tack, or bioadhesive behavior, but uncontrolled swelling may reduce dimensional stability or create hydration mismatch against a hydrophobic backing [91, 103, 104].

Quantitative sealing metrics should be reported as part of the device specification. Water‐vapor transmission rate (WVTR) is important because the skin‐contact environment must remain hydrated without maceration or dehydration, and CS‐PEO/fibrinogen composites have been reported with WVTR values compatible with wound‐healing environments [95]. In multilayer fibrous membranes, EC and sodium alginate networks can improve mechanical breaking strength while providing a hierarchical pore structure for controlled transport [85, 90]. However, hygroscopic alginate‐rich layers may swell and modify pore dimensions during storage or wear, reinforcing the need to match swelling behavior across the backing, reservoir, and hydrogel interface.

Safe and sustainable skin‐contact design also requires attention to microbiological quality, residual solvents, and crosslinking chemistry. Aqueous or ethanol‐compatible systems are preferable when they achieve adequate film formation and adhesion without leaving irritating residues [85, 98, 105]. CS production, cellulose dissolution and synthetic adhesive manufacture can all carry environmental burdens, so “natural” adhesion should not be assumed to be sustainable without considering extraction chemistry, solvent use and end‐of‐life behavior [69, 70, 99]. Therefore, the adhesive and sealing layers must provide a hermetic, zero‐gap fluidic manifold that remains compatible with hydrogel swelling, directional release, skin physiology and scalable manufacture.

## 5 Green Manufacturing and Scalable Fabrication

### 5.1 Why Manufacturing Must be Part of the Design Discussion

Manufacturing determines fiber diameter, porosity, residual solvent, drug distribution, protein stability, and dose reproducibility. Many microneedle studies demonstrate proof‐of‐concept insertion and release, but fewer report a process window that could support reproducible scale‐up [18, 106]. For a reservoir patch, the fabrication method must be compatible with each layer: hydrophobic backing, hydrophilic reservoir, diffusion‐control hydrogel, and microneedle interface.

### 5.2 Electrospinning, Centrifugal Spinning and PG

Electrospinning (ES), centrifugal spinning, and PG all generate polymer fibers by stretching a liquid jet, but the dominant driving force differs. ES uses a high‐voltage electric field to overcome surface tension and draw charged jets toward a collector, producing fine fibers but requiring suitable solution conductivity and often relatively low throughput. Centrifugal spinning uses rotational force to eject polymer solution from an orifice, stretching the jet as solvent evaporates, which can increase productivity and reduce dependence on electric fields. PG combines high‐speed rotation with applied gas pressure, so that centrifugal force and pressure‐driven flow act together to eject and stretch polymer jets into fibers [106, 107, 108]. For microneedle‐reservoir patches, this distinction matters because backing and reservoir layers require scalable fiber production, controlled porosity, and compatibility with drug‐loaded or biologic‐sensitive formulations.

### 5.3 PG for Fibrous Drug‐Delivery Reservoirs

PG is particularly relevant to fibrous drug‐delivery reservoirs because it can produce nano‐ to micrometer‐sized fibers, including hollow fibers from polymer solutions through the combined effects of vessel rotation, gas pressure, solution viscosity, and solvent evaporation [109]. In single‐layer PG, one polymer solution is processed into homogeneous fibers, which is suitable for fabricating backing layers, diffusion‐control mats, or single‐phase reservoir structures when one material can provide the required function. In contrast, core–sheath pressurized gyration separates two polymer streams into inner and outer domains, allowing one material to carry a hydrophilic drug or model payload while another provides hydrophobic protection, delayed wetting, or mechanical support [100]. This distinction maps directly onto reservoir‐patch design where single‐layer EC or CA fibers can support hydrophobic backing or structural layers, whereas PVP‐EC core–sheath fibers can combine a hydrophilic core for payload loading with an EC sheath for release modulation. Reported production rates for pressure‐spun PVP‐EC core‐sheath systems of ∼221 mg/min and fiber diameters of ∼2 µm illustrate why PG should be evaluated as a scalable reservoir‐fabrication route rather than only as a fiber‐forming curiosity [100]. Very recently, PG has also been shown to be energy‐efficient and scalable in both directions, from high‐throughput manufacture to compact battery‐ and solar‐powered platforms, enabling portable, off‐grid and potentially point‐of‐care fiber production [110, 111].

### 5.4 Green Solvent Systems

Green manufacturing requires matching each polymer to the mildest solvent system that still provides adequate solubility, viscosity, spinnability, and final device performance. Ethanol/water systems are relevant for EC and some cellulose‐derivative fiber processes, where they can reduce reliance on more hazardous organic solvents while preserving film‐ or fiber‐forming behavior [85]. Water is preferred for alginate, carboxymethyl cellulose (CMC), and some CMCS systems because it supports hydrogel formation and ionic crosslinking without organic solvent exposure [90, 91]. Dilute acetic acid is commonly used for CS and CS/PEO because protonation of chitosan amino groups is required for dissolution and fiber formation [93, 94]. By contrast, DMAc, THF, acetone, hexafluoro isopropanol (HFIP), and glutaraldehyde‐containing processes may still be useful for difficult polymers or high‐performance fibers, but they require stronger justification because of solvent toxicity, residual‐solvent control, and biological compatibility concerns [98, 100]. The key argument is not that every sustainable device must be purely aqueous, but that solvent choice should be reported, justified, and linked to drug stability, environmental burden, and manufacturability.

### 5.5 Hybrid Manufacturing, Lamination and Device Assembly

A clinically useful microneedle‐reservoir patch cannot be manufactured by optimizing a single microneedle array or a single fiber mat in isolation. It is a multilayer device in which the backing, reservoir, diffusion‐control layer, and HFMN interface must be assembled with controlled interfacial contact and reproducible dose geometry [21, 22, 32, 33, 66, 112, 113]. Hybrid manufacturing is therefore central to translation because each layer may require a different fabrication method, solvent environment, and drying history.

Fiber‐forming routes offer scalable options for reservoir and backing layers. ES can produce fine nanoscale fibers and drug‐loaded porous mats, but its low throughput and dependence on solution conductivity limit industrial translation [106, 114]. PG and pressure‐coupled infusion gyration use rotational and pressure‐driven forces to form micro‐ and nanofibers at higher production rates, with reported operating windows involving high rotation speeds, gas pressure, and controlled infusion [115, 116]. These approaches are particularly relevant when gram‐scale or higher‐throughput reservoir production is required [108, 117].

Hybrid fiber systems can generate functional gradients within a patch. PG combined with ES has been used to create multilayer or targeted fiber deposits, where a high‐throughput process supplies the bulk structure and a precision deposition step localizes bioactive material [106, 108]. Core–sheath PG can place hydrophilic cargo within a polymer core while using a hydrophobic sheath to regulate wetting and release [100]. Such architectures are attractive for microneedle‐reservoir systems because they allow hydrophilic model cargo or protein‐like payloads to be separated from moisture‐sensitive backing layers.

Micro‐molding, casting, photopolymerization, and 3D‐printed molds remain essential for the HFMN interface. Polydimethylsiloxane (PDMS), teflon, and printed molds allow reproducible needle geometry, while casting and drying conditions determine tip filling, residual water, and baseplate flexibility [101]. For reservoir‐coupled patches, the manufacturing challenge is to align and laminate the fiber reservoir onto the microneedle baseplate without creating air gaps or lateral leakage. Layer‐by‐layer assembly and polyelectrolyte complexation can also create diffusion‐control membranes under mild conditions, which is relevant for alginate, CS, and other bio‐derived networks [118, 119, 120].

Scale‐up introduces variables that are rarely tested in proof‐of‐concept work. Biopolymer molecular‐weight variation can alter viscosity, fiber diameter, and drug distribution [89, 93, 106, 121]. Ambient humidity and temperature influence ES, solvent removal, and hydrogel swelling, while residual solvents such as chloroform, DMAc, or THF require stringent control or replacement by water/ethanol systems [98, 122]. Microbiological‐control requirements must also be considered during device assembly because any selected sterilization, decontamination, or aseptic‐manufacturing strategy may affect polymer structure and biomacromolecule stability [72, 123]. Lyophilization should be considered separately as a drying and stabilization process because it can also alter reservoir structure and cargo stability. Hybrid manufacturing should therefore be introduced at the design stage, rather than after material optimization, so that dose uniformity, lamination, sealing, drying, microbiological quality, and packaging are developed as one manufacturing pathway.

## 6 Model Macromolecules: FITC‐Dextran and Other Surrogates

### 6.1 Why are Model Macromolecules Used?

Model macromolecules are used to determine whether a delivery system can load, release, and transport large hydrophilic species before evaluation with an active therapeutic. FITC‐dextran is particularly useful because fluorescent labeling permits sensitive quantification and spatial visualization across defined molecular‐weight ranges. However, fluorescent dextrans represent only one class of surrogate. Protein models such as BSA and ovalbumin provide complementary information because their globular structures more closely approximate protein therapeutics, whereas labeled peptides or FITC‐insulin can be used when spatial localization of a peptide‐sized molecule is required [21, 31, 48, 124]. Surrogate selection should therefore reflect the experimental question, including molecular size, structure, charge, analytical detectability, and whether the intended endpoint is release, diffusion, skin deposition, or permeation.

These model systems achieve their function through measurable chemical or optical signals. FITC‐dextran is covalently labeled fluorescein, allowing the transported mass to be quantified by fluorescence spectroscopy and visualized in skin sections by fluorescence or confocal microscopy. Different dextran molecular weights can be used to test size‐dependent transport through hydrogel networks and skin, as shown by the higher permeation of lower‐molecular‐weight FITC‐dextran compared with larger dextran species in HFMN studies [31]. BSA and ovalbumin provide protein‐sized structures that better approximate globular macromolecules, while FITC‐insulin can provide a closer peptide‐specific signal when the aim is to visualize insulin distribution. However, fluorescence confirms molecular presence, not biological function and therefore, model macromolecules prove physical release and transport but do not prove folding, receptor binding, pharmacokinetics, or pharmacodynamic activity [31, 123, 124].

### 6.2 Molecular Weight Matching versus Biological Equivalence

Molecular‐weight matching is not biological equivalence. FITC‐dextran 10 kDa can act as an insulin‐sized hydrophilic transport surrogate, but it does not model insulin folding, aggregation, receptor binding, glucose‐lowering activity, or pharmacodynamics [31, 124]. FITC‐dextran transport through HFMNs decreases with molecular weight, with FD10 showing higher permeation than FD150 under comparable conditions, supporting its value for size‐dependent transport analysis [31]. Figure 7 illustrates fluorescent insulin transport through skin after microneedle application, emphasizing the additional evidence needed for therapeutic claims.

**FIGURE 7:**
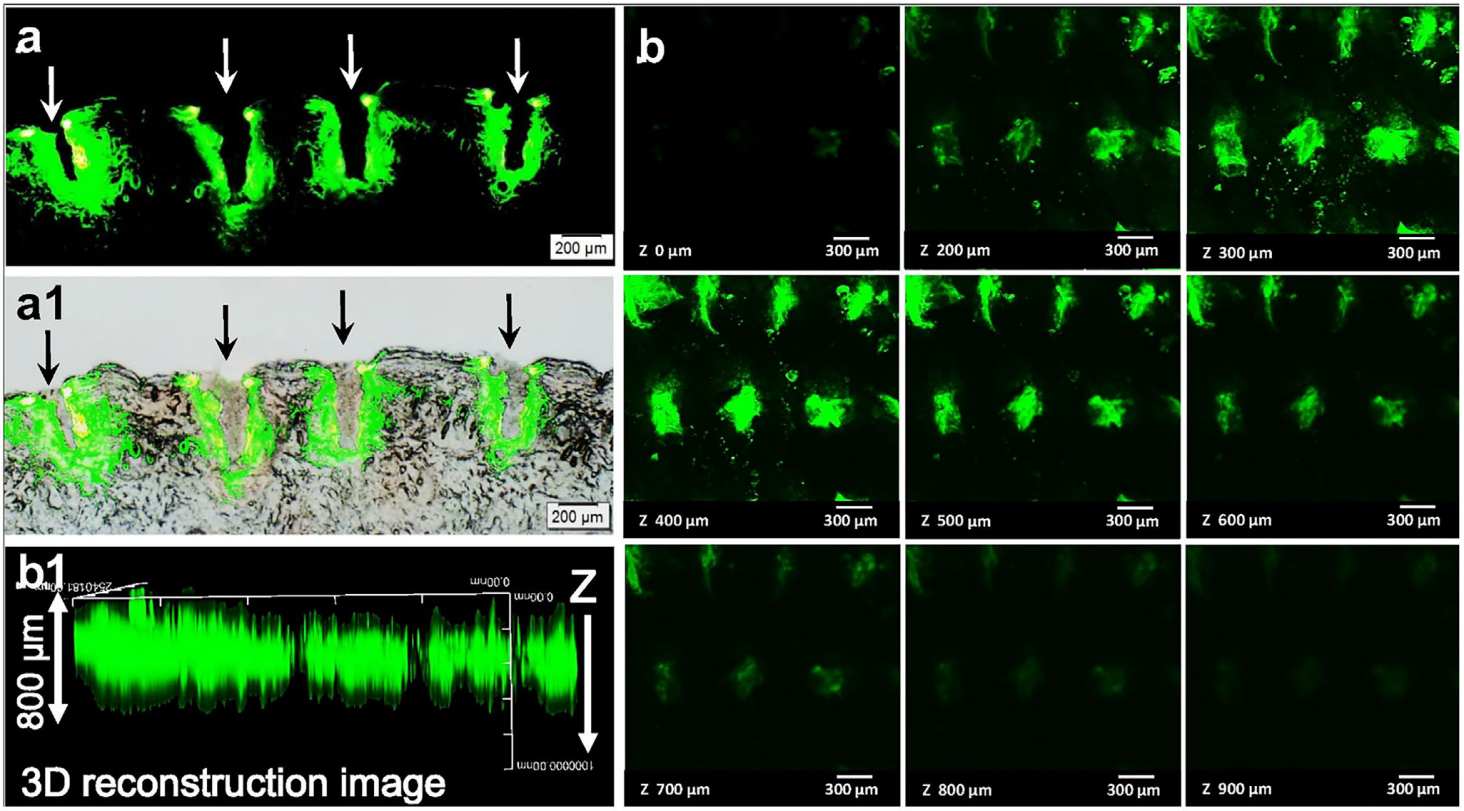
FITC‐insulin delivery visualized after microneedle insertion. Fluorescence microscopy and 3D reconstruction show depth‐resolved delivery of FITC‐labeled insulin through skin puncture sites. Poly‐gamma‐glutamic acid microneedles with a supporting structure design as a potential tool for transdermal delivery of insulin (Copyright 2015 Acta Materialia Inc. Published by Elsevier Ltd.) [124].

This distinction is particularly important for FITC‐dextran 10 kDa. Its molecular weight can be closer to insulin than smaller dyes, but its linear polysaccharide architecture, hydrodynamic behavior, and charge distribution differ from folded peptide and protein therapeutics [31, 123]. Therefore, FD10 is best described as an insulin‐sized physical transport surrogate, not an insulin surrogate in a pharmacological sense.

### 6.3 What Evidence Is Acceptable at Each Stage

Early material development can be supported by fluorescence release, Franz diffusion, skin deposition and recovery studies using FITC‐dextran or FITC‐BSA. However, therapeutic claims require the real drug: stability during fabrication and storage, retained bioactivity, pharmacokinetics, pharmacodynamics, and safety [2, 18, 123, 125]. For insulin‐like claims, evidence should progress from physical transport of FD10 to release of insulin, retention of secondary structure, receptor‐related activity, and finally glucose‐lowering performance in a relevant model [5, 6, 126].

### 6.4 A Proposed Reporting Framework for Model‐Cargo Studies

Model‐cargo studies would be more comparable if they adopted a minimum reporting framework that links the selected surrogate to the claim being made. At present, studies report fluorescence release, skin deposition, permeation, or cumulative delivery using different molecular weights, skin models, receptor media, and recovery methods, which make direct comparison difficult [31, 48, 124]. Future studies should specify the model molecule, molecular weight, hydrodynamic size where available, charge, concentration, loading method, release medium, skin model, receptor conditions, recovery efficiency, and analytical calibration. More importantly, authors should explicitly state whether the experiment demonstrates physical release, skin deposition, transdermal permeation, or therapeutic activity. Fluorescent signal within skin confirms the presence of a labeled molecule, but it does not confirm that an active protein remains folded, biologically active, or therapeutically effective [31, 123, 124]. This distinction would reduce overclaiming and make FITC‐dextran, FITC‐BSA, and FITC‐insulin studies more useful for translational comparison.

### 6.5 From Model Cargo to Therapeutic Validation

Translational validation of HFMN‐reservoir patches should follow an evidence ladder rather than move directly from fluorescence release to therapeutic claims. Early‐stage studies often use rhodamine 6G, FITC‐dextran, FITC‐BSA, or ovalbumin because these models are safer, quantifiable, and available over defined molecular‐weight ranges [21, 31, 48]. Such molecules are useful for measuring loading, release, diffusion, and skin deposition, but they primarily validate physical transport rather than therapeutic function [31].

The first tier of evidence is reservoir release and hydrogel diffusion. FITC‐dextran of different molecular weights allows researchers to test whether mesh size and swelling permit transport of peptide‐ or protein‐sized hydrophilic molecules [31, 127]. Conductive or iontophoretic microneedle platforms have also been used to enhance dextran transport and demonstrate electrically assisted macromolecular movement [128, 129]. However, dextran is a linear polysaccharide, whereas insulin, albumin, ovalbumin, and antibodies are folded or globular biomolecules, so molecular‐weight matching does not establish biological equivalence [31].

The second tier is skin deposition and permeation. Franz diffusion cells quantify cumulative permeation and flux, while confocal microscopy or in vivo imaging can reveal depth‐resolved deposition of fluorescent payloads [31, 48]. Hollow microneedle studies with FITC‐dextran further show that intradermal administration volume influences pharmacokinetic profiles, reinforcing that delivery geometry and dose volume cannot be ignored [130]. Fluorescent signal must nevertheless be interpreted cautiously: it confirms localization of a labeled molecule, but not necessarily folding, receptor binding, or pharmacological effect.

The third tier is real‐drug stability and retained structure. Insulin, peptides and proteins can denature or aggregate during solvent exposure, drying, electric‐field assisted processing, pressurized fiber formation or sterilization [123, 131]. Circular dichroism, FTIR, High‐Performance Liquid Chromatography (HPLC), Size‐Exclusion Chromatography (SEC), and Sodium Dodecyl Sulfate‐Polyacrylamide Gel Electrophoresis (SDS‐PAGE) are therefore needed to confirm secondary structure, chemical integrity, and aggregation state after fabrication and storage [123, 131]. Model protein delivery, such as BSA or ovalbumin, should be followed by real therapeutic release and a bioactivity assay, because mass recovery alone does not prove efficacy [21, 52, 132].

The final tiers are pharmacokinetics, pharmacodynamics, and immune response. For insulin‐like systems, plasma insulin levels must be linked to glucose reduction and relative pharmacological availability [125]. For vaccines or protein antigens, validation must include antigen stability, lymphatic targeting and immune activation rather than fluorescence deposition alone [50, 132, 133]. The design rule is that model cargo should be used to de‐risk transport, but translational claims require evidence that the real therapeutic remains stable, bioactive, and effective in an appropriate biological model.

### 6.6 Beyond Fluorescent Surrogates: Therapeutic Macromolecule Validation

Fluorescent model molecules are indispensable during early microneedle‐reservoir development because they allow rapid quantification of release, skin deposition and microchannel‐mediated transport [134]. FITC‐dextran is particularly useful for assessing size‐dependent movement of hydrophilic species, but it does not reproduce the folding, aggregation tendency, receptor binding, or biological potency of peptide and protein therapeutics [31, 123]. Globular models such as BSA and ovalbumin approximate protein size more closely than linear dextran, but mass recovery or fluorescence still cannot prove retained antigenicity or immune function [21, 52].

The use of FITC‐insulin provides a closer peptide‐specific visual marker, but labeling chemistry can alter charge, hydrodynamic size, and receptor interaction. Therefore, identity verification and purity assessment are required when fluorescent insulin is used as a surrogate for therapeutic insulin [124]. This distinction is central to avoiding fluorescence fallacy, as a strong dermal signal demonstrates the presence of a labeled molecule but does not demonstrate that an active biologic has survived fabrication, storage, release, and skin transport [123, 124].

Biomacromolecule instability can be triggered by solvent exposure, drying, shear, UV irradiation, pH changes, adsorption to polymers, and sterilization conditions [123, 131]. Protein and peptide validation should therefore include analytical methods that answer different questions, such as HPLC and size‐exclusion chromatography for purity, recovery, and aggregation; SDS‐PAGE and mass spectrometry for degradation and identity; circular dichroism or FTIR for secondary structure; enzyme‐linked immunosorbent assay (ELISA) or receptor‐binding assays for antigenicity; and cell‐based or in vivo assays for functional potency [123, 131, 132].

For insulin‐like delivery claims, the evidence ladder should progress from model transport to therapeutic activity. FITC‐dextran or FITC‐BSA can demonstrate physical transport; FITC‐insulin can indicate peptide localization; real insulin release must then be paired with structural recovery, aggregation analysis, and pharmacodynamic glucose‐lowering evidence in relevant models [125, 126, 128]. Ultimately, a translational microneedle‐reservoir patch must prove both transport and biological function, because sustainable manufacturing has little clinical value if it compromises the activity of the biomacromolecule [123, 131].

## 7 Characterization Standards for Microneedle‐Reservoir Patches

### 7.1 Material and Structural Characterization

A reservoir patch should be characterized at material, layer, and device levels. Scanning Electron Microscopy (SEM) should quantify fiber diameter, needle height, base width, tip radius and interspacing; porosity, thickness, contact angle and water‐vapor transmission should be reported for backing and reservoir layers. FTIR, x‐ray diffraction (XRD), Differential Scanning Calorimetry (DSC), and Thermogravimetric Analysis (TGA) should be used when crosslinking, crystallinity, or thermal stability are relevant [89, 121, 135]. PG has produced 10 wt.% EC fibers with an average diameter of approximately 623 nm at 6000 rpm and 0.3 MPa [135], while gravity‐driven ultrahigh‐speed ES has generated porous EC fibers with specific surface areas of up to 16.9 m^2^ g^−1^ [88].

### 7.2 Microneedle Performance

Microneedle testing must distinguish whole‐array force from force per needle. Compression testing, fracture force, height reduction, parafilm insertion, porcine or human skin insertion, dye staining, and optical coherence tomography provide different levels of evidence and should not be treated as interchangeable [60, 61, 62]. Figure 8 shows mechanical and insertion testing for CMCS‐SFP/OPL microneedles, where a reported breaking force of 88 mN per needle provides a useful benchmark for natural‐polymer HFMNs [68].

**FIGURE 8:**
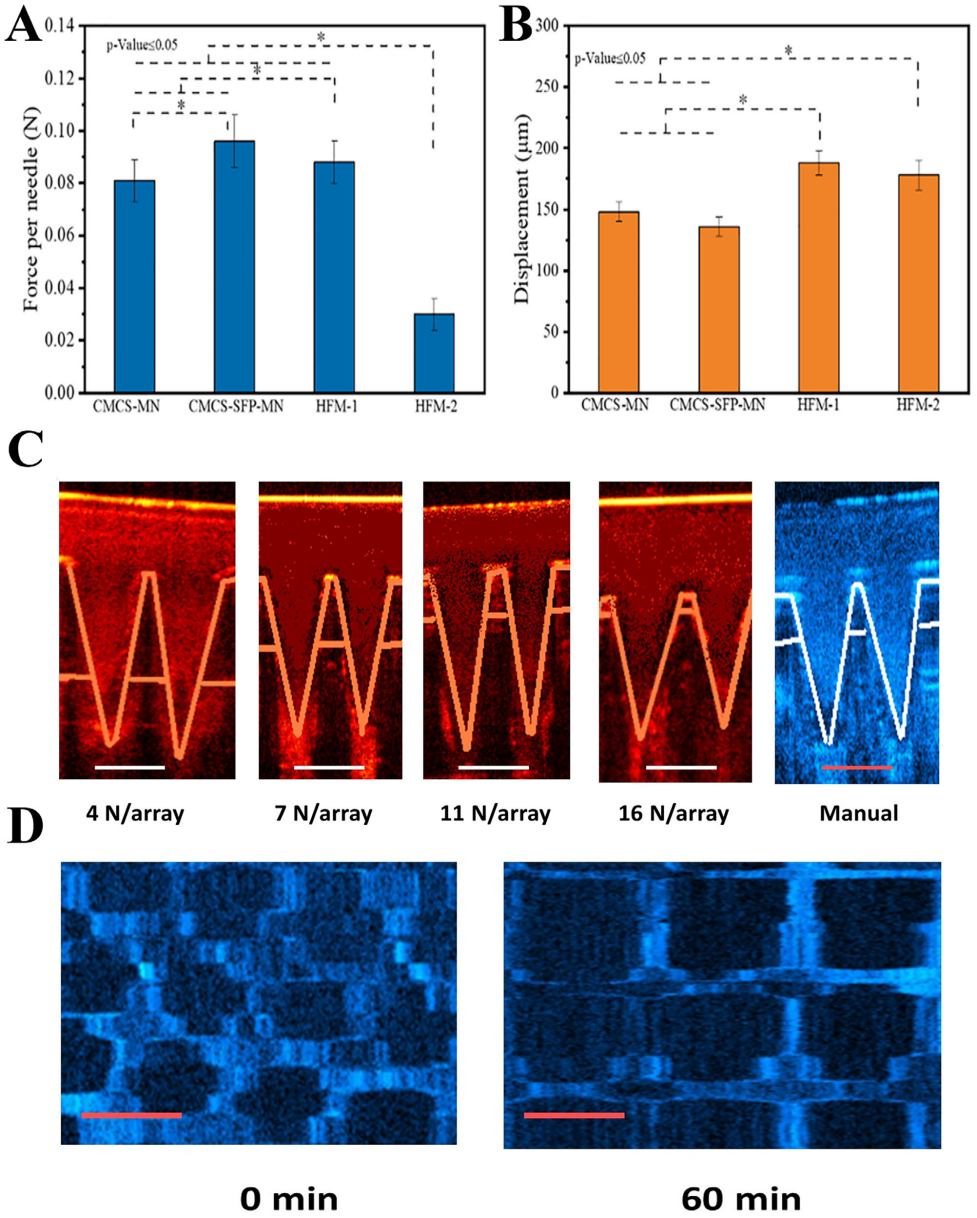
Mechanical performance and skin insertion of HFMNs. (A) Breaking force per microneedle and (B) Maximum displacement at breaking force for CMCS‐SFP/OPL hydrogel microneedles. From Hydrogel‐based microneedles of chitosan derivatives for drug delivery (Copyright 2022 Elsevier B.V.) [68]. (C) Optical coherence tomography image of a HFMN array following insertion into excised neonatal porcine skin, and (D) OCT visualization of the resulting skin micro conduit immediately following microneedle application. Scale bars in C and D represent 300 µm. Panels C and D from Hydrogel‐Forming Microneedles Prepared from “Super Swelling” Polymers Combined with Lyophilized Wafers for Transdermal Drug Delivery under the terms of the Creative Commons Attribution License [21].

### 7.3 Release and Permeation Testing

Release kinetics should be interpreted through a hierarchy of techniques. In vitro release studies, typically performed in Phosphate‐Buffered Saline (PBS) or another receptor medium, are useful for comparing reservoir formulations, estimating burst release and fitting kinetic models such as Higuchi or Korsmeyer‐Peppas, but they do not include the SC barrier. Franz diffusion‐cell studies using synthetic membranes, porcine skin, or human cadaver skin add a permeation barrier and allow cumulative permeation, flux, and lag time to be measured under defined donor and receptor conditions [21, 31, 33, 48]. Fluorescence microscopy and confocal imaging then provide spatial evidence of skin deposition and penetration depth, while OCT can confirm insertion without destroying the sample [21, 60, 136]. Finally, in vivo studies are required when systemic pharmacokinetics, pharmacodynamics, immune response, or glucose‐lowering activity is claimed, because only living systems include blood flow, tissue clearance, and biological feedback [2, 50, 125, 128, 132]. Therefore, each technique answers a different question like in vitro release shows formulation behavior, Franz diffusion shows barrier transport, imaging shows localization, and in vivo testing shows biological performance.

Previous microneedle and reservoir studies also differ substantially in the type of release data they report. Some studies focus on total cumulative permeation, such as the higher 24 h transport of FD10 compared with FD150 through HFMN‐treated skin, while others emphasize release percentage, flux, lag time, or fitted kinetic models [31, 48]. For example, PVA/CMC dissolving microneedles containing catechin released 87 ± 5% over 14 h, whereas HFMN‐reservoir systems have reported delivery of 44 mg ibuprofen sodium and 1.24 mg ovalbumin over 24 h, reflecting a different reservoir‐controlled mechanism rather than a directly comparable release percentage [21, 26, 137]. Core–sheath PVP/EC pressure‐spun fibers have also shown pronounced early release, with 35%–73% ampicillin release within the first hour depending on formulation, highlighting how fibrous reservoir architecture can influence burst behavior [100]. These differences show that future studies should report burst release, sustained‐release duration, release rate, cumulative mass, percentage release, flux, and model‐fitting parameters together, rather than relying on a single release percentage.

### 7.4 Biocompatibility and Safety

Biocompatibility and local safety should be evaluated at the level of the complete microneedle‐reservoir device rather than inferred only from the known biocompatibility of individual polymers. Cytotoxicity, hemocompatibility, irritation, residual solvent, microbiological‐control processes and skin‐barrier recovery may all change after crosslinking, drug loading, drying, lamination and storage [18, 61, 71, 72]. Transepidermal water loss (TEWL), erythema, edema, inflammatory markers and repeated‐application studies are especially relevant for chronic delivery of insulin‐like or peptide therapeutics, because these patches may need to be applied repeatedly to the same or adjacent skin sites [138]. Therefore, a polymer that appears cytocompatible in extract testing should not be described as device‐safe unless the final loaded and stored patch has also been assessed under conditions that reflect its intended use.

Microbiological quality remains an important but incompletely standardized aspect of microneedle translation. Because microneedles transiently disrupt the SC, the finished product requires an appropriate microbiological specification, but terminal sterility should not be assumed to be necessary for every microneedle product. Current regulatory thinking supports product‐specific assessment of infection risk, with the appropriate specification determined by factors including product design, microbial burden, administration setting, application site, target population and clinical context [71]. A low‐bioburden specification may therefore be appropriate for some products, whereas sterilization or aseptic manufacture may remain necessary when justified by the risk assessment [71]. Where sterilization is required, gamma irradiation, ethylene oxide, heat, or other processes must be evaluated for their effects on polymer integrity, swelling behavior, residual processing agents, and drug stability [18, 72, 123].

### 7.5 Quality‐by‐Design Thinking

Quality‐by‐design (QbD) is a systematic development approach in which the desired product performance is defined first, and formulation and process variables are then controlled to achieve that performance reproducibly. For microneedle‐reservoir patches, QbD means linking critical material attributes, such as polymer molecular weight, concentration, crosslink density, solvent ratio, residual water, and payload stability, with critical process parameters, such as rpm, pressure, voltage, mold filling, drying time, crosslinking conditions, humidity, lamination pressure, and sealing method [139]. These variables are then connected to critical quality attributes, including needle integrity, insertion depth, swelling ratio, drug loading, burst release, flux, microbiological specification, residual solvent, and retained bioactivity. This is particularly important for peptide and protein delivery because a small change in polymer concentration or crosslink density can simultaneously alter insertion strength, swelling rate, and release kinetics.

QbD is useful because insertion, swelling, and release are mechanistically connected rather than independent outcomes. Increasing polymer concentration or crosslink density may improve dry mechanical strength and reduce height reduction during compression, but it can also decrease mesh size, slow swelling, and reduce macromolecule diffusion. Conversely, reducing crosslink density may increase swelling and flux but weaken the hydrated array and increase the risk of deformation during wear [21, 23, 56, 139, 140]. Humidity and drying conditions influence residual water and tip sharpness, whereas spinning speed, pressure, and solvent ratio influence fiber diameter and porosity, and lamination pressure and edge sealing influence reservoir contact and dose uniformity [18, 100, 106, 139]. A QbD‐guided study should therefore define acceptable ranges for force per needle, insertion depth, swelling ratio, burst release, cumulative permeation, residual solvent, and bioactivity, then use formulation and manufacturing variables to keep the device inside that design space. This approach would make microneedle‐reservoir patches more comparable between laboratories and more credible for translation. Table 5 therefore provides a minimum reporting framework for the structural, mechanical, release, and safety parameters required to compare microneedle‐reservoir patch studies across laboratories.

**TABLE 5:** Minimum reporting framework for microneedle‐reservoir patch studies.

Domain	Minimum measurements	Why it matters	Examples of useful values	Ref.
Geometry and structure	Needle height/base/tip, interspacing, fiber diameter, porosity, layer thickness	Controls insertion and reservoir transport	PLGA microneedles ∼415 µm height	[48]
Mechanical performance	Fracture force, height reduction, insertion depth, whole‐array and per‐needle force	Determines safe insertion margin	CMCS‐SFP/OPL 88 mN/needle; Gantrez height reduction 12% at 50 mN/needle	[29, 68]
Release and permeation	Release %, burst %, lag time, flux, cumulative permeation, skin model, sink conditions	Allows comparison across systems	FD10 HFMN permeation higher than FD150	[31]
Biological and translational safety	Cell viability, irritation, TEWL, microbiological specification/bioburden, residual solvent, storage stability	Moves evidence from material to device level	CMCS/OPL >75% viability; TEWL recovery can occur within hours to days depending on occlusion	[68, 71, 138]

### 7.6 Analytical Methods for Biomacromolecule Stability and Activity

For hydrophilic biomacromolecules, successful permeation is not equivalent to successful therapy. Insulin, peptides, enzymes, recombinant proteins and antibodies may lose function during polymer mixing, drying, micro‐molding, ES, PG, sterilization or storage [123, 131]. Analytical characterization must therefore move beyond total mass release and establish identity, purity, aggregation state, secondary structure, and biological activity.

FTIR spectroscopy is useful for monitoring polymer–drug interactions, hydrogen bonding, polyelectrolyte complexation and chemical changes after processing [119]. Circular dichroism is more directly relevant for protein therapeutics because it probes secondary structure and can show whether insulin or peptide‐like payloads retain alpha‐helical and beta‐sheet content after fabrication or triggered release [123, 131]. Fluorescence spectroscopy and confocal microscopy are valuable for spatial tracking of labeled cargo, but their outputs should be reported as transport evidence, not as proof of retained bioactivity [31, 48].

Chromatographic and electrophoretic methods provide stronger evidence of molecular integrity. HPLC or RP‐HPLC can quantify drug content, percentage recovery, and degradation products, while size‐exclusion chromatography can reveal aggregation or fragmentation in protein‐containing reservoirs. SDS‐PAGE can detect gross fragmentation or aggregation of protein payloads, and mass spectrometry can provide higher‐resolution identification of degradation products where required for regulatory development [123, 131]. These methods should be applied after fabrication, drying, any selected sterilization or decontamination process, and accelerated storage rather than only to the starting solution.

Functional assays are essential because structural preservation does not always guarantee biological activity. ELISA can confirm retained antigenicity or specific protein recognition, receptor‐binding assays can confirm target engagement, and cell‐based assays can test whether released peptides or proteins retain their biological function [123, 132]. For insulin patches, in vivo glucose reduction and plasma insulin measurement remain the key pharmacodynamic and pharmacokinetic endpoints [125, 128]. For vaccine or antigen‐delivery systems, immune readouts such as antibody titer, dendritic‐cell activation, or lymph node targeting are more relevant than total antigen release alone [50, 132, 133].

Safety‐related analytical tests should be treated as part of biomacromolecule quality, not as a final regulatory add‐on. Endotoxin testing, cytotoxicity, residual solvent analysis, skin irritation, TEWL recovery, and histology provide complementary information on whether the final device is safe after processing and storage [15]. Therefore, biomacromolecule integrity and retained function should be defined as critical quality attributes, measured at every major manufacturing step and reported alongside permeation data.

### 7.7 Skin Models and Testing Hierarchy

The translation of HFMN‐reservoir platforms requires a testing hierarchy that separates mechanical insertion, passive release, skin permeation, dermal deposition, and biological response [9, 10]. Artificial membranes are useful for high‐throughput in vitro release testing because they reduce biological variability and allow controlled comparison of formulations. Strat‐M is frequently used because its multilayered structure contains components that approximate the lipid organization of skin, while Skin‐Parallel Artificial Membrane Permeability Assay (PAMPA) has shown strong correlation with mammalian skin for selected finite‐dose permeation studies [141, 142]. However, these models cannot reproduce the viscoelastic deformation, microvascular clearance, or interstitial‐fluid uptake that govern HFMN swelling [21, 23, 29, 56].

Ex vivo biological skin remains essential for permeation and deposition studies. Excised human skin is the most clinically relevant in vitro barrier because it preserves the structure of the SC and viable epidermis, but donor variability, limited supply, and ethical constraints reduce routine availability [7, 10]. Porcine skin is widely used as a practical alternative because its SC thickness, follicle density, and general architecture resemble human skin more closely than most small‐animal models [10]. Nevertheless, ex vivo skin lacks blood flow and immune function, so it may underestimate clearance and cannot prove systemic pharmacokinetics or pharmacodynamics [9, 11].

Reconstructed human epidermis and full‐thickness engineered skin models provide a scalable and ethically favorable route for screening irritation, barrier disruption, and disease‐specific permeability [10]. Pathological models, including filaggrin‐deficient or inflammation‐mimicking constructs, may be valuable for dermal biologic delivery, but they are generally more permeable and mechanically less robust than native skin [10, 11]. For microneedles, this matters because a model that predicts molecular diffusion may still fail to predict insertion reliability, swelling pressure, or tissue recovery.

In vivo studies are required when claims involve systemic exposure, pharmacodynamic activity, or immune response. Rodents are convenient for insulin and vaccine screening, but their thin skin can overestimate delivery compared with human skin [42, 124]. Minipigs provide a more clinically relevant large‐animal model, although cost and ethical restrictions limit routine use [10]. Microneedle‐based testing is also moving beyond insertion and permeation studies toward point‐of‐care diagnostic platforms, where the microneedle interface can access interstitial fluid and support minimally invasive biomarker analysis [143]. Hydrogel microneedle assays extend this concept further by combining interstitial‐fluid sampling with electrochemical aptamer sensing and colorimetric pH monitoring, showing that swollen hydrogel arrays can function as both sampling interfaces and analytical devices [144]. Final human studies should combine pharmacokinetics or pharmacodynamics with local safety metrics such as erythema, itching, tenderness, and TEWL recovery, because microneedle‐created pores can reseal over time ranging from hours to more than a day depending on geometry and occlusion [138].

## 8 Translation

### 8.1 Dose Loading and Dose Accuracy

For biologics and insulin‐like molecules, dose accuracy is not optional, yet the literature remains inconsistent in how dose control is reported. Some studies report drug loading or encapsulation efficiency; others report percentage release, cumulative permeation, fluorescence intensity, or pharmacodynamic response, and these endpoints are not interchangeable [31, 48, 124]. A patch may insert successfully and release fluorescent cargo but still fail translationally if loading varies between patches, reservoir‐to‐microneedle contact is incomplete, drug migrates between layers during storage, or only a fraction of the loaded dose becomes bioavailable [18, 28]. This issue is particularly important for insulin because both under‐delivery and over‐delivery can be clinically significant. Future studies should therefore report absolute drug loading, loading uniformity, dose recovery, patch‐to‐patch variation, delivered mass, residual drug after application and, for real therapeutics, retained bioactivity and pharmacodynamic response [18, 61, 123, 125]. This would shift the field from demonstrating that material platforms can transport model cargo to proving that complete devices can deliver therapeutically meaningful and reproducible doses.

### 8.2 Microbiological Control, Packaging and Storage

Microbiological control, packaging and storage are decisive for HFMN‐reservoir patches because the device must remain sharp, mechanically competent, and chemically stable while meeting an appropriate microbiological specification before use. Although microneedles transiently disrupt the SC, sterility should not be assumed to be a universal requirement for all microneedle products [18, 71]. Current regulatory thinking instead supports a product‐specific assessment of infection risk, with the finished product potentially specified as either sterile or low bioburden according to its design, microbial burden, administration setting, application site, target population and clinical context [71]. Where sterilization or aseptic manufacture is required, the selected process must be compatible with both the polymeric device and the biomacromolecular payload. Gamma irradiation, ethylene oxide, UV treatment, aseptic processing and heat‐based methods can each affect polymer chain integrity, swelling behavior, residual sterilant levels and biomacromolecule stability [18, 72, 123]. Microbiological‐control strategy should therefore be defined during product development rather than imposed after formulation.

Packaging must control water activity, oxygen exposure and mechanical damage. Hydrogel‐forming systems can absorb moisture during storage, altering tip sharpness, crosslink density and swelling kinetics [24]. Sealed foil pouches, desiccants and humidity‐controlled storage are therefore essential where hygroscopic reservoirs or hydrogel microneedles are used [18, 24, 145]. Storage claims should report real‐time and accelerated stability, mechanical integrity after storage, residual drug content, retained bioactivity, and post‐storage insertion performance.

Long‐term stability should distinguish preservation of drug content from preservation of therapeutic quality. For peptide and protein reservoirs, unchanged total drug content does not exclude aggregation, fragmentation, oxidation, deamidation, or conformational changes that reduce biological activity [123, 131]. Real‐time and accelerated stability studies should therefore assess identity, purity, aggregation state and retained potency together with reservoir morphology, HFMN mechanical integrity, insertion performance and delivered dose [123, 145]. Shelf‐life should be assigned to the assembled and packaged product rather than inferred from stability of the isolated drug, reservoir or microneedle components.

### 8.3 Manufacturing Reproducibility and Quality Control

Manufacturing reproducibility is a central barrier to translating microneedle‐reservoir patches because small changes in mold filling, drying, fiber diameter, reservoir loading, or lamination pressure can change delivered dose. Micro‐molding is widely used for polymeric microneedle arrays, but incomplete mold filling, high solution viscosity, and air entrapment can generate defective tips or variable needle height [61, 101]. Fiber‐based reservoirs introduce additional sources of variability, including polymer molecular weight, solution viscosity, humidity, residual solvent, and drug distribution across the mat [106, 114].

Reservoir coupling introduces an additional assembly and load‐transfer challenge that is less pronounced in simpler dissolving microneedle systems. Lyophilized wafers and soft or wax‐like polymeric reservoirs can be mechanically fragile or deformable, so the drug‐containing layer cannot necessarily transmit the force required for microneedle insertion without damage [21, 22, 66]. The backing and applicator must therefore protect the reservoir from fracture, excessive compression, or lateral displacement while transferring sufficient force to the HFMN baseplate for reproducible skin penetration. Backing stiffness, reservoir thickness, interfacial contact, lamination pressure and applicator mechanics must consequently be considered together. Insertion performance should be evaluated using the fully assembled patch, rather than the isolated microneedle array alone, and should be accompanied by assessment of reservoir integrity and reservoir‐to‐HFMN contact after application [21, 22, 81, 146].

Translation from laboratory fabrication to good manufacturing practice (GMP) production requires formulation and process variables to be converted into defined incoming‐material specifications, in‐process controls and finished‐product release criteria [18, 61]. Incoming material controls should address polymer identity, molecular weight or substitution characteristics where relevant, moisture content, and biomacromolecule identity and potency. In‐process controls should include solution concentration and viscosity, mold filling, reservoir mass, layer thickness, drying conditions, lamination, alignment, humidity and residual solvent [61, 106, 139]. Finished‐product release should assess microneedle geometry and integrity, insertion performance, reservoir content uniformity, delivered dose, residual solvent and water, microbiological specification, and retained biomacromolecule activity [18, 61, 71]. In‐line imaging, gravimetric loading checks and content‐uniformity assays can support lot‐to‐lot control. Release specifications should therefore apply to the fully assembled patch rather than to its individual components in isolation.

### 8.4 Regulatory Uncertainty and Drug‐Device Combination Products

Microneedle‐reservoir patches sit at the intersection of formulation, device engineering and biologic therapy, which complicates regulatory classification. A patch that contains a biologic, a microneedle array, a reservoir, adhesive layers and a backing may need evidence for both medicinal product quality and device performance [18, 61]. For macromolecules, the regulatory package must address identity, potency, microbiological quality and infection risk, residual solvent, delivered dose, device failure, skin irritation and storage stability rather than only release percentage.

For a drug‐device combination product, pharmaceutical and device performance cannot be evaluated independently. Drug‐related critical quality attributes include identity, purity, potency, reservoir drug content and content uniformity, whereas device‐related attributes include microneedle geometry, mechanical integrity, insertion performance, reservoir‐to‐HFMN coupling and applicator function [18, 61, 139]. The clinically relevant output is the dose delivered by the assembled product rather than reservoir loading alone. Stability and failure testing should therefore determine whether changes in the drug, reservoir, microneedle array, backing, or interlayer interfaces alter insertion or dose delivery during the intended shelf‐life [18, 61]. Regulatory development should link formulation controls, device performance, and conditions of use within a common target product profile.

The combination‐product challenge is amplified by target use. A vaccine patch requires immunogenicity and antigen stability, an insulin patch requires pharmacokinetics and glucose‐lowering pharmacodynamics, and an interstitial‐fluid sampling patch requires analytical accuracy and biocompatibility [50, 125, 143]. Future submissions will be strengthened by target‐product‐profile‐driven development, where the intended clinical use defines the required dose, wear time, microbiological‐control strategy, applicator design, and release specification before materials are selected [3, 18, 28].

### 8.5 Sustainability versus Clinical Practicality

Sustainability must be balanced against clinical practicality. Natural polymers, aqueous processing and biodegradable components are valuable only if they also support dose accuracy, appropriate microbiological control, storage, reproducible insertion and patient usability [69, 70, 99]. Conversely, synthetic or semi‐synthetic systems may be clinically practical because they are mechanically robust and stable, but they may require improvement in solvent use, manufacturing energy, or disposal [18, 98].

The strongest translational position is therefore not to replace every synthetic component with a natural one, but to design a complete patch in which solvent choice, crosslinking chemistry, fiber production, packaging, shelf‐life and end‐of‐life handling are justified by performance data [99, 108]. Economic translation also matters: microneedle systems must offer clear benefits in adherence, efficacy, storage or dose control to justify GMP manufacturing and commercial scale‐up. Sustainable microneedle‐reservoir patches should therefore be judged by integrated clinical and environmental performance rather than by polymer origin alone.

### 8.6 Clinical Usability, Patient Handling and Commercial Translation

The clinical success of microneedle‐reservoir systems will depend on patient handling as much as on material performance. Microneedles are attractive because they are minimally invasive and can reduce the pain, anxiety, and tissue damage associated with repeated hypodermic injections [14, 15, 16, 81]. This advantage is particularly relevant for chronic diseases, where simplified self‐administration and reduced dosing frequency could improve adherence [3, 6, 81]. However, usability cannot be assumed: elderly patients, children, and users with reduced dexterity may apply patches with variable force, angle, or residence time, producing inconsistent insertion and drug exposure.

Dose confidence is a critical translational requirement because regulatory agencies have low tolerance for variable exposure, especially for potent biologics and peptide therapeutics. Applicator‐assisted insertion, spring‐loaded systems, and tactile or visual feedback mechanisms can reduce user variability by standardizing insertion force and confirming successful application. Packaging must also protect fragile micro‐scale arrays from humidity and mechanical shock during transport, while desiccants and foil‐laminate barriers may be needed for hygroscopic hydrogel or dissolving matrices [18, 24, 145].

Commercial translation remains constrained by manufacturing cost, scale‐up yield, and quality‐control requirements. High‐fidelity core–shell or reservoir‐coupled designs can improve loading and release control, but they can increase production and assembly complexity relative to simpler dissolving or coated arrays because the backing, reservoir and HFMN baseplate must remain mechanically integrated during storage, insertion and wear. Nevertheless, microneedle vaccines and self‐administered patches may become cost‐effective when they reduce healthcare‐worker time, cold‐chain burden, sharps disposal and clinic visits. For sustainable devices, biodegradability and reduced sharps waste are attractive, but they must be balanced against mechanical integrity, storage stability and manufacturability [99].

Therefore, clinically viable microneedle‐reservoir patches should combine a simple user workflow, fail‐safe applicator feedback, humidity‐protective packaging, validated dose uniformity and scalable manufacturing so that patient convenience does not compromise therapeutic precision.

## 9 Outlook: Target‐Product‐Profile‐Driven Design Rules for Microneedle‐Reservoir Patches

The next stage of microneedle‐reservoir patch development should begin with a target product profile rather than with a preferred polymer. An insulin or glucagon‐like peptide‐1 (GLP‐1)‐patch requires accurate dose delivery, rapid or basal pharmacodynamic control, retained peptide structure, and protection against dose dumping [125, 128, 147, 148, 149]. A vaccine or antigen patch requires antigen stability, skin immune‐cell targeting, an appropriate microbiological specification, and storage without loss of immunogenicity [50, 132, 145]. A local dermal biologic or wound‐healing patch requires conformability, controlled residence time, and compatibility with inflamed or damaged tissue [54, 150, 151]. Recent HA hydrogel microneedles incorporating MXene and a triplet mRNA formulation have demonstrated controlled macromolecular release together with enhanced angiogenesis and tissue regeneration in diabetic wounds, illustrating the extension of microneedle platforms toward locally acting regenerative macromolecule therapies [150].

For sustained peptide/protein depot patches, the reservoir and diffusion‐control layer should be designed around release duration, loading capacity, and protein stability rather than needle volume [22, 28]. For interstitial‐fluid sampling or theranostic patches, the priority shifts toward analyte recovery, swelling kinetics, sensor integration, and patient comfort rather than maximal drug loading [75, 143, 144]. These examples show why one “best” microneedle material does not exist; rather, the material system must be chosen according to dose, duration, target tissue, storage requirement, and manufacturing route. Future microneedle‐reservoir patches are likely to converge with smart theranostic technologies, where the same skin‐interfacing platform can support controlled delivery, interstitial‐fluid sensing, wound stimulation, or regenerative therapy [25, 143, 144]. Electrical microneedles and microneedle‐mediated cell therapy extend this concept beyond conventional drug release by introducing active stimulation, tissue repair, and living‐cell delivery into minimally invasive patch platforms [151, 152, 153, 154]. These examples reinforce that the next generation of microneedle‐reservoir devices should be designed as integrated therapeutic systems rather than as isolated arrays. Bioinspired control of interfacial transport may also inform future reservoir design. Outside transdermal delivery, liquid‐gating topological‐gradient microfluidic systems have shown how interface geometry and wetting can be engineered to regulate fluid passage and directional transport [155]. These principles have not yet been established for HFMN‐reservoir patches, but they provide a relevant engineering basis for investigating interfaces that regulate reservoir hydration, directional liquid entry, and resistance to leakage.

Seven design rules emerge. First, define the target product profile before polymer selection [3]. Second, separate the functions of insertion, swelling, drug storage, diffusion control, adhesion, and backing protection within a multilayer architecture [21, 22]. Third, match hydrogel mesh size and crosslink density to the hydrodynamic dimensions of the intended cargo [23, 31, 56]. Fourth, treat biomacromolecule integrity and bioactivity as critical quality attributes rather than secondary confirmation. Fifth, design solvent choice, drying, lamination, and the microbiological‐control strategy together so that manufacturing does not compromise payload integrity or finished‐product safety [18, 71, 98]. Sixth, standardized insertion, swelling, release, permeation, dose recovery, and storage data to enable cross‐study comparison [18, 61, 139]. Seventh, support sustainability claims with evidence on solvent use, energy, stability, packaging and disposal, not only natural polymer origin [69, 99, 108, 110, 111].

## 10 Conclusions

HFMN‐reservoir patches offer a promising route to overcome the mismatch between the therapeutic importance of hydrophilic biomacromolecules and the limitations of injection‐based delivery. Their principal advantage is the separation of skin insertion, swelling‐mediated conduit formation, drug storage, diffusion control, and environmental protection into distinct but integrated functions. Material selection should increasingly favor renewable and biocompatible systems, including alginate, CS and cellulose‐based materials such as EC, CA and other functional cellulose derivatives, while recognizing that natural origin alone does not establish sustainability. Solvent choice, crosslinking chemistry, process yield, stability, microbiological control, and end‐of‐life considerations remain decisive. Recent fiber‐manufacturing approaches, particularly PG, which combines centrifugal rotation with gas pressure to generate fibers rapidly and at high throughput, offer a scalable route for producing fibrous layers for multilayer reservoir systems using appropriately selected aqueous or ethanol‐rich formulations. FITC‐dextran and related model macromolecules remain useful for establishing transport mechanisms, but therapeutic translation requires confirmation using active biomacromolecules with retained stability and bioactivity. Progress will therefore depend on integrating natural‐material selection, scalable manufacturing, multilayer architecture, quantitative release performance and regulatory requirements from the earliest stages of device development.

## Author Contributions

**Siddharth Mohanty**: writing – original draft, writing – review and editing, conceptualization, investigation, methodology, validation, visualization, data curation. **Mehmet Onur Aydogdu**: writing – original draft, writing – review and editing, supervision, validation, conceptualization, project administration. **Sijia Kang**: writing – original draft, writing – review and editing, validation, conceptualization. **Xinbo Li**: writing – review and editing, writing – original draft, validation, conceptualization. **Mohan Edirisinghe**: funding acquisition, writing – original draft, writing – review and editing, supervision, project administration, validation, conceptualization.

## Conflicts of Interest

The authors declare no conflicts of interest.

## Data Availability

Data sharing not applicable to this article as no datasets were generated or analysed during the current study.
